# Deflated preconditioned conjugate gradient method for solving single-step BLUP models efficiently

**DOI:** 10.1186/s12711-018-0429-3

**Published:** 2018-11-03

**Authors:** Jérémie Vandenplas, Herwin Eding, Mario P. L. Calus, Cornelis Vuik

**Affiliations:** 1 Animal Breeding and Genomics Centre, Wageningen UR Livestock Research, P.O. Box 338, 6700 AH Wageningen, The Netherlands; 2 CRV BV, Wassenaarweg 20, 6843 NW Arnhem, The Netherlands; 3 DIAM, TU Delft, Van Mourik Broekmanweg 6, 2628 XE Delft, The Netherlands

## Abstract

**Background:**

The single-step single nucleotide polymorphism best linear unbiased prediction (ssSNPBLUP) method, such as single-step genomic BLUP (ssGBLUP), simultaneously analyses phenotypic, pedigree, and genomic information of genotyped and non-genotyped animals. In contrast to ssGBLUP, SNP effects are fitted explicitly as random effects in the ssSNPBLUP model. Similarly, principal components associated with the genomic information can be fitted explicitly as random effects in a single-step principal component BLUP (ssPCBLUP) model to remove noise in genomic information. Single-step genomic BLUP is solved efficiently by using the preconditioned conjugate gradient (PCG) method. Unfortunately, convergence issues have been reported when solving ssSNPBLUP by using PCG. Poor convergence may be linked with poor spectral condition numbers of the preconditioned coefficient matrices of ssSNPBLUP. These condition numbers, and thus convergence, could be improved through the deflated PCG (DPCG) method, which is a two-level PCG method for ill-conditioned linear systems. Therefore, the first aim of this study was to compare the properties of the preconditioned coefficient matrices of ssGBLUP and ssSNPBLUP, and to document convergence patterns that are obtained with the PCG method. The second aim was to implement and test the efficiency of a DPCG method for solving ssSNPBLUP and ssPCBLUP.

**Results:**

For two dairy cattle datasets, the smallest eigenvalues obtained for ssSNPBLUP (ssPCBLUP) and ssGBLUP, both solved with the PCG method, were similar. However, the largest eigenvalues obtained for ssSNPBLUP and ssPCBLUP were larger than those for ssGBLUP, which resulted in larger condition numbers and in slow convergence for both systems solved by the PCG method. Different implementations of the DPCG method led to smaller condition numbers, and faster convergence for ssSNPBLUP and for ssPCBLUP, by deflating the largest unfavourable eigenvalues.

**Conclusions:**

Poor convergence of ssSNPBLUP and ssPCBLUP when solved by the PCG method are related to larger eigenvalues and larger condition numbers in comparison to ssGBLUP. These convergence issues were solved with a DPCG method that annihilates the effect of the largest unfavourable eigenvalues of the preconditioned coefficient matrix of ssSNPBLUP and of ssPCBLUP on the convergence of the PCG method. It resulted in a convergence pattern, at least, similar to that of ssGBLUP.

**Electronic supplementary material:**

The online version of this article (10.1186/s12711-018-0429-3) contains supplementary material, which is available to authorized users.

## Background

In general, genomic data for livestock animals include several thousand single nucleotide polymorphisms (SNPs), which are used in genetic evaluations to obtain genomic estimated breeding values [1–3]. Currently, the method of choice that simultaneously combines phenotypic and pedigree information of genotyped and non-genotyped animals with genomic information of genotyped animals is the so-called single-step genomic best linear unbiased prediction (ssGBLUP) [3]. ssGBLUP includes genomic information by combining genomic and pedigree relationships into a combined genomic-pedigree relationship matrix [3–5]. However, a major inconvenience of ssGBLUP is that the inverse of a dense genomic relationship matrix (${\mathbf{G}}$) is required, which can be computed up to approximately 100,000 genotyped animals on current computers [6]. Thus, some methods were proposed to approximate the inverse of ${\mathbf{G}}$, such as the algorithm for proven and young animals (APY) [6], or to compute its inverse implicitly based on singular value decomposition (SVD) [7] or on the Woodbury decomposition [8]. Another approach to avoid the computation of the inverse of ${\mathbf{G}}$, or even ${\mathbf{G}}$ itself, is to fit the SNP effects explicitly, or principal components obtained from a SVD of the genotype matrix, as random effects in the model. Several equivalent models were proposed in the literature that enable simultaneous modelling of genotyped and non-genotyped animals as in ssGBLUP [2, 7, 9–13]. Equivalent models that directly estimate SNP effects as random effects [2, 9–13] will hereafter be referred to as single-step SNPBLUP (ssSNPBLUP). It has been suggested that the dimension of SNP-based models can be considerably reduced by applying random regression to principal components (PC) of the SNP genotypes, and that the remaining noise of genomic information can be ignored [14]. To our knowledge, a linear system of equations of single-step principal component BLUP (ssPCBLUP) has never been solved with the PCG method for large datasets.

The ssGBLUP, ssSNPBLUP and ssPCBLUP models have linear systems of equations with sparse and symmetric positive (semi-)definite (SPSD) coefficient matrices. Thus, the preconditioned conjugate gradient (PCG) method is the primary choice as iterative solver for solving linear systems of ssGBLUP, ssSNPBLUP [11, 15–17], and of ssPCBLUP. The PCG method belongs to the family of conjugate gradient (CG) methods that are a realization of an orthogonal projection technique onto the Krylov subspace, which is generated by the initial residual and the system matrix (e.g., the preconditioned coefficient matrix) to which the CG method is applied [17]. The convergence rate of CG methods is bounded as a function of the spectral condition number of the system matrix, which is the ratio between the largest and smallest eigenvalues of the system matrix [17]. Preconditioning ensures faster convergence of the PCG method, compared to the CG method. Unfortunately, in contrast to ssGBLUP, convergence issues with the PCG method applied to ssSNPBLUP have been reported [11, 18], which we have also experienced in our initial analyses. Furthermore, we experienced similar convergence issues with ssPCBLUP in our initial analyses.

Taskinen et al. [11] suggested that convergence problems may be due to a poor spectral condition number of the system matrix of ssSNPBLUP. Thus, to achieve faster convergence, improvement of this spectral condition number is needed and can be obtained through methods that have been developed for ill-conditioned linear systems of equations. One such method is the deflated PCG method, which is a two-level PCG method for ill-conditioned linear systems [19–21]. The DPCG method has resulted in good performance in other contexts than genetic evaluations [22–24], and possesses interesting properties, such as its relatively easy implementation in current software based on a PCG method and its favourable properties for parallel computing [22]. To our knowledge, the DPCG method has never been applied in linear mixed models, whether for genetic evaluations or other purposes. Thus, the first aim of this study was to compare the properties of the system matrices of ssGBLUP of the ssSNPBLUP model that was proposed by Mantysaari and Stranden [13], and to relate this to observed convergence patterns obtained with the PCG method. Our second aim was to implement the DPCG method and test its feasibility for solving ssSNPBLUP in large genetic evaluation models, and its re-parametrization into a ssPCBLUP model.

## Methods

The first part of this section describes the ssSNPBLUP model that was proposed by Mantysaari and Stranden [13] and its re-parametrization into a ssPCBLUP model. The second part describes the CG, PCG, and DPCG methods. The last part describes the datasets used for comparing the properties of the system matrices of the different models, and for testing the DPCG method.

### A ssSNPBLUP model

In this study, we investigate the ssSNPBLUP model that was proposed by Mantysaari and Stranden [13] and is similar to the so-called hybrid model proposed by Fernando et al. [10]. This ssSNPBLUP model fits three types of additive genetic effects: SNP and residual polygenic effects for genotyped animals, and additive genetic effects for non-genotyped animals. Originally derived as a univariate ssSNPBLUP model, we (readily) extended this to a multivariate ssSNPBLUP model for $t$ traits. In the following, ${\mathbf{I}}_{t}$ is an identity matrix with size equal to the number of traits $t$, and the subscripts $g$ and $n$ refer to genotyped and non-genotyped animals, respectively. A standard multivariate mixed model for ssSNPBLUP can be written as:

1 $${\mathbf{y}} = {\mathbf{Xb}} + \left[ {\begin{array}{*{20}c} {{\mathbf{W}}_{n} } & \mathbf{0} & \mathbf{0} \\ \mathbf{0} & {{\mathbf{W}}_{g} } & {{\mathbf{W}}_{g} {\mathbf{M}}_{z} } \\ \end{array} } \right]\left[ {\begin{array}{*{20}c} {{\mathbf{u}}_{n} } \\ {{\mathbf{a}}_{g} } \\ {\mathbf{g}} \\ \end{array} } \right] + {\mathbf{e}},$$

where ${\mathbf{y}}$ is the vector of records, ${\mathbf{b}}$ is the vector of fixed effects, ${\mathbf{u}}_{n}$ is the vector of additive genetic effects for non-genotyped animals, ${\mathbf{a}}_{g}$ is the vector of residual polygenic effects for genotyped animals, ${\mathbf{g}}$ is the vector of SNP effects, and ${\mathbf{e}}$ is the vector of residuals. The matrices ${\mathbf{X}}$, ${\mathbf{W}}_{n}$, and ${\mathbf{W}}_{g}$ are incidence matrices relating records to the corresponding effects. Without loss of generality, the matrix ${\mathbf{Z}}$ contains the SNP genotypes (coded as 0 for one homozygous genotype, 1 for the heterozygous genotype, or 2 for the alternate homozygous genotype) centered by their observed means, and ${\mathbf{M}}_{z} = {\mathbf{I}}_{t} \otimes {\mathbf{Z}}$.

Additive genetic effects for the genotyped animals for $t$ traits, ${\mathbf{u}}_{g}$, can be computed as ${\mathbf{u}}_{g} = {\mathbf{a}}_{g} + {\mathbf{M}}_{z} {\mathbf{g}}$. We assume a multivariate normal distribution for the additive genetic effects ${\mathbf{u}}_{n}$, the residual polygenic effects ${\mathbf{a}}_{g}$, and the SNP effects ${\mathbf{g}}$, with a mean equal to zero and covariance matrix ${\varvec{\Sigma}}$, $$\left[ {\begin{array}{*{20}c} {{\mathbf{u}}_{n} } \\ {{\mathbf{a}}_{g} } \\ {\mathbf{g}} \\ \end{array} } \right]\sim{\kern 1pt} MVN\left( {\left[ {\begin{array}{*{20}c} \mathbf{0} \\ \mathbf{0} \\ \mathbf{0} \\ \end{array} } \right],{\varvec{\Sigma}}} \right)$$. The inverse of ${\varvec{\Sigma}}$ required for the mixed model equations associated with Eq. (1), can be derived from the inverse of the (co)variance matrix associated with the vector $$\left[ {\begin{array}{*{20}c} {{\mathbf{u}}_{n} } \\ {{\mathbf{u}}_{g} } \\ {\mathbf{g}} \\ \end{array} } \right]$$, $$var\left( {\begin{array}{*{20}c} {{\mathbf{u}}_{n} } \\ {{\mathbf{u}}_{g} } \\ {\mathbf{g}} \\ \end{array} } \right)$$, proposed by Liu et al. [12] as follows [13]:

$$ \begin{aligned} {\varvec{\Sigma}}^{ - 1} & = \left( {var\left( {\left[ {\begin{array}{*{20}c} {{\mathbf{u}}_{n} } \\ {{\mathbf{a}}_{g} } \\ {\mathbf{g}} \\ \end{array} } \right]} \right)} \right)^{ - 1} = \left( {var\left( {\left[ {\begin{array}{*{20}c} {\mathbf{I}} & {\mathbf{0}} & {\mathbf{0}} \\ {\mathbf{0}} & {\mathbf{I}} & { - {\mathbf{M}}_{z} } \\ {\mathbf{0}} & {\mathbf{0}} & {\mathbf{I}} \\ \end{array} } \right]\left[ {\begin{array}{*{20}c} {{\mathbf{u}}_{n} } \\ {{\mathbf{u}}_{g} } \\ {\mathbf{g}} \\ \end{array} } \right]} \right)} \right)^{ - 1} \\ & = \left( {\left[ {\begin{array}{*{20}c} {\mathbf{I}} & {\mathbf{0}} & {\mathbf{0}} \\ {\mathbf{0}} & {\mathbf{I}} & { - {\mathbf{M}}_{z} } \\ {\mathbf{0}} & {\mathbf{0}} & {\mathbf{I}} \\ \end{array} } \right]var\left( {\left[ {\begin{array}{*{20}c} {{\mathbf{u}}_{n} } \\ {{\mathbf{u}}_{g} } \\ {\mathbf{g}} \\ \end{array} } \right]} \right)\left[ {\begin{array}{*{20}c} {\mathbf{I}} & {\mathbf{0}} & {\mathbf{0}} \\ {\mathbf{0}} & {\mathbf{I}} & {\mathbf{0}} \\ {\mathbf{0}} & { - {\mathbf{M}}_{z}^{\varvec{'}} } & {\mathbf{I}} \\ \end{array} } \right]} \right)^{ - 1} \\ & = \left[ {\begin{array}{*{20}c} {\mathbf{I}} & {\mathbf{0}} & {\mathbf{0}} \\ {\mathbf{0}} & {\mathbf{I}} & {\mathbf{0}} \\ {\mathbf{0}} & { - {\mathbf{M}}_{z}^{\varvec{'}} } & {\mathbf{I}} \\ \end{array} } \right]^{ - 1} \left( {var\left( {\left[ {\begin{array}{*{20}c} {{\mathbf{u}}_{n} } \\ {{\mathbf{u}}_{g} } \\ {\mathbf{g}} \\ \end{array} } \right]} \right)} \right)^{ - 1} \left[ {\begin{array}{*{20}c} {\mathbf{I}} & {\mathbf{0}} & {\mathbf{0}} \\ {\mathbf{0}} & {\mathbf{I}} & { - {\mathbf{M}}_{z} } \\ {\mathbf{0}} & {\mathbf{0}} & {\mathbf{I}} \\ \end{array} } \right]^{ - 1} \\ & = \left[ {\begin{array}{*{20}c} {\mathbf{I}} & {\mathbf{0}} & {\mathbf{0}} \\ {\mathbf{0}} & {\mathbf{I}} & {\mathbf{0}} \\ {\mathbf{0}} & {{\mathbf{M}}_{z}^{\varvec{'}} } & {\mathbf{I}} \\ \end{array} } \right]\left( {var\left( {\left[ {\begin{array}{*{20}c} {{\mathbf{u}}_{n} } \\ {{\mathbf{u}}_{g} } \\ {\mathbf{g}} \\ \end{array} } \right]} \right)} \right)^{ - 1} \left[ {\begin{array}{*{20}c} {\mathbf{I}} & {\mathbf{0}} & {\mathbf{0}} \\ {\mathbf{0}} & {\mathbf{I}} & {{\mathbf{M}}_{z} } \\ {\mathbf{0}} & {\mathbf{0}} & {\mathbf{I}} \\ \end{array} } \right] \\ & = \left[ {\begin{array}{*{20}c} {\mathbf{I}} & {\mathbf{0}} & {\mathbf{0}} \\ {\mathbf{0}} & {\mathbf{I}} & {\mathbf{0}} \\ {\mathbf{0}} & {{\mathbf{M}}_{z}^{\varvec{'}} } & {\mathbf{I}} \\ \end{array} } \right]\left[ {\begin{array}{*{20}c} {{\mathbf{G}}_{0}^{ - 1} \otimes {\mathbf{A}}^{nn} } & {{\mathbf{G}}_{0}^{ - 1} \otimes {\mathbf{A}}^{ng} } & {\mathbf{0}} \\ {{\mathbf{G}}_{0}^{ - 1} \otimes {\mathbf{A}}^{gn} } & {\begin{array}{*{20}c} {{\mathbf{G}}_{0}^{ - 1} \otimes {\mathbf{A}}^{gg} + } \\ {\left( {\frac{1}{w} - 1} \right){\mathbf{G}}_{0}^{ - 1} \otimes {\mathbf{A}}_{gg}^{ - 1} } \\ \end{array} } & { - \frac{1}{w}{\mathbf{G}}_{0}^{ - 1} \otimes {\mathbf{A}}_{gg}^{ - 1} {\mathbf{Z}}} \\ {\mathbf{0}} & { - \frac{1}{w}{\mathbf{G}}_{0}^{ - 1} \otimes {\mathbf{Z^{\prime}A}}_{gg}^{ - 1} } & {\frac{1}{w}{\mathbf{G}}_{0}^{ - 1} \otimes {\mathbf{Z^{\prime}A}}_{gg}^{ - 1} {\mathbf{Z}} + \frac{m}{1 - w}{\mathbf{G}}_{0}^{ - 1} \otimes {\mathbf{I}}} \\ \end{array} } \right]\left[ {\begin{array}{*{20}c} {\mathbf{I}} & {\mathbf{0}} & {\mathbf{0}} \\ {\mathbf{0}} & {\mathbf{I}} & {{\mathbf{M}}_{z} } \\ {\mathbf{0}} & {\mathbf{0}} & {\mathbf{I}} \\ \end{array} } \right] \\ & = \left[ {\begin{array}{*{20}c} {{\mathbf{G}}_{0}^{ - 1} \otimes {\mathbf{A}}^{nn} } & {{\mathbf{G}}_{0}^{ - 1} \otimes {\mathbf{A}}^{ng} } & {{\mathbf{G}}_{0}^{ - 1} \otimes {\mathbf{A}}^{ng} {\mathbf{Z}}} \\ {{\mathbf{G}}_{0}^{ - 1} \otimes {\mathbf{A}}^{gn} } & {{\mathbf{G}}_{0}^{ - 1} \otimes {\mathbf{A}}^{gg} + \left( {\frac{1}{w} - 1} \right){\mathbf{G}}_{0}^{ - 1} \otimes {\mathbf{A}}_{gg}^{ - 1} } & {{\mathbf{G}}_{0}^{ - 1} \otimes \left( {{\mathbf{A}}^{gg} - {\mathbf{A}}_{gg}^{ - 1} } \right){\mathbf{Z}}} \\ {{\mathbf{G}}_{0}^{ - 1} \otimes {\mathbf{Z^{\prime}A}}^{gn} } & {{\mathbf{G}}_{0}^{ - 1} \otimes {\mathbf{Z^{\prime}}}\left( {{\mathbf{A}}^{gg} - {\mathbf{A}}_{gg}^{ - 1} } \right)} & {{\mathbf{G}}_{0}^{ - 1} \otimes {\mathbf{Z^{\prime}}}\left( {{\mathbf{A}}^{gg} - {\mathbf{A}}_{gg}^{ - 1} } \right){\mathbf{Z}} + \frac{m}{1 - w}{\mathbf{G}}_{0}^{ - 1} \otimes {\mathbf{I}}} \\ \end{array} } \right], \\ \end{aligned} $$

where $$\left[ {\begin{array}{*{20}c} {{\mathbf{u}}_{n} } \\ {{\mathbf{a}}_{g} } \\ {\mathbf{g}} \\ \end{array} } \right] = \left[ {\begin{array}{*{20}c} {{\mathbf{u}}_{n} } \\ {{\mathbf{u}}_{g} - {\mathbf{M}}_{z} {\mathbf{g}}} \\ {\mathbf{g}} \\ \end{array} } \right] = \left[ {\begin{array}{*{20}c} {\mathbf{I}} & \mathbf{0} & \mathbf{0} \\ \mathbf{0} & {\mathbf{I}} & { - {\mathbf{M}}_{z} } \\ \mathbf{0} & \mathbf{0} & {\mathbf{I}} \\ \end{array} } \right]\left[ {\begin{array}{*{20}c} {{\mathbf{u}}_{n} } \\ {{\mathbf{u}}_{g} } \\ {\mathbf{g}} \\ \end{array} } \right]$$, $$\left[ {\begin{array}{*{20}c} {\mathbf{I}} & \mathbf{0} & \mathbf{0} \\ \mathbf{0} & {\mathbf{I}} & { - {\mathbf{M}}_{z} } \\ \mathbf{0} & \mathbf{0} & {\mathbf{I}} \\ \end{array} } \right]^{ - 1} = \left[ {\begin{array}{*{20}c} {\mathbf{I}} & \mathbf{0} & \mathbf{0} \\ \mathbf{0} & {\mathbf{I}} & {{\mathbf{M}}_{z} } \\ \mathbf{0} & \mathbf{0} & {\mathbf{I}} \\ \end{array} } \right]$$, ${\mathbf{G}}_{0}^{ - 1}$ is the inverse of the (co)variance matrix among traits, $${\mathbf{A}}^{ - 1} = \left[ {\begin{array}{*{20}c} {{\mathbf{A}}^{nn} } & {{\mathbf{A}}^{ng} } \\ {{\mathbf{A}}^{gn} } & {{\mathbf{A}}^{gg} } \\ \end{array} } \right]$$ is the inverse of the pedigree relationship matrix, ${\mathbf{A}}_{gg}$ is the pedigree relationship matrix among genotyped animals, $w$ is the proportion of variance (due to additive genetic effects) considered as residual polygenic effects, and $m = 2\sum p_{i} \left( {1 - p_{i} } \right)$ with $p_{i}$ being the allele frequency of the $i$ th SNP, and such that $var\left( {\mathbf{g}} \right) = \frac{1 - w}{m}{\mathbf{G}}_{0} \otimes {\mathbf{I}}$.

Knowing that ${\mathbf{A}}_{gg}^{ - 1} = {\mathbf{A}}^{gg} - {\mathbf{A}}^{gn} \left( {{\mathbf{A}}^{nn} } \right)^{ - 1} {\mathbf{A}}^{ng}$, and after some algebra to avoid the computation of ${\mathbf{A}}_{gg}^{ - 1}$ to form ${\varvec{\Sigma}}^{ - 1}$, we obtain:

$$\begin{aligned} {\varvec{\Sigma}}^{ - 1} & = \left[ {\begin{array}{*{20}c} {{\mathbf{G}}_{0}^{ - 1} \otimes {\mathbf{A}}^{nn} } & {{\mathbf{G}}_{0}^{ - 1} \otimes {\mathbf{A}}^{ng} } & {{\mathbf{G}}_{0}^{ - 1} \otimes {\mathbf{A}}^{ng} {\mathbf{Z}}} \\ {{\mathbf{G}}_{0}^{ - 1} \otimes {\mathbf{A}}^{gn} } & {\frac{1}{w}{\mathbf{G}}_{0}^{ - 1} \otimes {\mathbf{A}}^{gg} + \left( {1 - \frac{1}{w}} \right){\mathbf{G}}_{0}^{ - 1} \otimes {\mathbf{Q}}} & {{\mathbf{G}}_{0}^{ - 1} \otimes {\mathbf{QZ}}} \\ {{\mathbf{G}}_{0}^{ - 1} \otimes {\mathbf{Z^{\prime}A}}^{gn} } & {{\mathbf{G}}_{0}^{ - 1} \otimes {\mathbf{Z^{\prime}Q}}} & {{\mathbf{G}}_{0}^{ - 1} \otimes {\mathbf{Z^{\prime}QZ}} + \frac{m}{1 - w}{\mathbf{G}}_{0}^{ - 1} \otimes {\mathbf{I}}} \\ \end{array} } \right] \\ & = \left[ {\begin{array}{*{20}c} {{\varvec{\Sigma}}^{11} } & {{\varvec{\Sigma}}^{12} } & {{\varvec{\Sigma}}^{13} } \\ {{\varvec{\Sigma}}^{21} } & {{\varvec{\Sigma}}^{22} } & {{\varvec{\Sigma}}^{23} } \\ {{\varvec{\Sigma}}^{31} } & {{\varvec{\Sigma}}^{32} } & {{\varvec{\Sigma}}^{33} } \\ \end{array} } \right], \\ \end{aligned}$$

where $${\mathbf{Q}} = {\mathbf{A}}^{gn} \left( {{\mathbf{A}}^{nn} } \right)^{ - 1} {\mathbf{A}}^{ng}$$.

The linear system of mixed model equations of ssSNPBLUP is as follows:

2 $$\left[ {\begin{array}{*{20}l} {{\mathbf{X^{\prime}R}}^{ - 1} {\mathbf{X}}} \hfill & {{\mathbf{X}}_{n}^{'} {\mathbf{R}}_{n}^{ - 1} {\mathbf{W}}_{n} } \hfill & {{\mathbf{X}}_{g}^{'} {\mathbf{R}}_{g}^{ - 1} {\mathbf{W}}_{g} } \hfill & {{\mathbf{X}}_{g}^{'} {\mathbf{R}}_{g}^{ - 1} {\mathbf{W}}_{g} {\mathbf{M}}_{z} } \hfill \\ {{\mathbf{W}}_{n}^{'} {\mathbf{R}}_{n}^{ - 1} {\mathbf{X}}_{n} } \hfill & {{\mathbf{W}}_{n}^{'} {\mathbf{R}}_{n}^{ - 1} {\mathbf{W}}_{n} + {\varvec{\Sigma}}^{11} } \hfill & {{\varvec{\Sigma}}^{12} } \hfill & {{\varvec{\Sigma}}^{13} } \hfill \\ {{\mathbf{W}}_{g}^{'} {\mathbf{R}}_{g}^{ - 1} {\mathbf{X}}_{g} } \hfill & {{\varvec{\Sigma}}^{21} } \hfill & {{\mathbf{W}}_{g}^{'} {\mathbf{R}}_{g}^{ - 1 } {\mathbf{W}}_{g} + {\varvec{\Sigma}}^{22} } \hfill & {{\mathbf{W}}_{g}^{'} {\mathbf{R}}_{g}^{ - 1} {\mathbf{W}}_{g} {\mathbf{M}}_{z} + {\varvec{\Sigma}}^{23} } \hfill \\ {{\mathbf{M}}_{z}^{\varvec{'}} {\mathbf{W}}_{g}^{'} {\mathbf{R}}_{g}^{ - 1} {\mathbf{X}}_{g} } \hfill & {{\varvec{\Sigma}}^{31} } \hfill & {{\mathbf{M}}_{z}^{\varvec{'}} {\mathbf{W}}_{g}^{'} {\mathbf{R}}_{g}^{ - 1} {\mathbf{W}}_{g} + {\varvec{\Sigma}}^{32} } \hfill & {{\mathbf{M}}_{z}^{\varvec{'}} {\mathbf{W}}_{g}^{'} {\mathbf{R}}_{g}^{ - 1} {\mathbf{W}}_{g} {\mathbf{M}}_{z} + {\varvec{\Sigma}}^{33} } \hfill \\ \end{array} } \right]\left[ {\begin{array}{*{20}c} {{\hat{\mathbf{b}}}} \\ {{\hat{\mathbf{u}}}_{n} } \\ {{\hat{\mathbf{a}}}_{g} } \\ {{\hat{\mathbf{g}}}} \\ \end{array} } \right] = \left[ {\begin{array}{*{20}c} {{\mathbf{X^{\prime}R}}^{ - 1} {\mathbf{y}}} \\ {{\mathbf{W}}_{n}^{'} {\mathbf{R}}_{n}^{ - 1} {\mathbf{y}}} \\ {{\mathbf{W}}_{g}^{'} {\mathbf{R}}_{g}^{ - 1} {\mathbf{y}}} \\ {{\mathbf{M}}_{z}^{\varvec{'}} {\mathbf{W}}_{g}^{'} {\mathbf{R}}_{g}^{ - 1} {\mathbf{y}}} \\ \end{array} } \right],$$

where $${\mathbf{R}}^{ - 1} = \left[ {\begin{array}{*{20}c} {{\mathbf{R}}_{n}^{ - 1} } & \mathbf{0} \\ \mathbf{0} & {{\mathbf{R}}_{g}^{ - 1} } \\ \end{array} } \right]$$ is the inverse of the residual (co)variance structure matrix.

This ssSNBLUP model is equivalent to the following ssGBLUP model:

3 $${\mathbf{y}} = {\mathbf{Xb}} + \left[ {\begin{array}{*{20}c} {{\mathbf{W}}_{n} } & \mathbf{0} \\ \mathbf{0} & {{\mathbf{W}}_{g} } \\ \end{array} } \right]\left[ {\begin{array}{*{20}c} {{\mathbf{u}}_{n} } \\ {{\mathbf{u}}_{g} } \\ \end{array} } \right] + {\mathbf{e}} ,$$

with $$var\left( {\begin{array}{*{20}c} {{\mathbf{u}}_{n} } \\ {{\mathbf{u}}_{g} } \\ \end{array} } \right) = {\mathbf{G}}_{0}^{ - 1} \otimes \left[ {\begin{array}{*{20}c} {{\mathbf{A}}^{nn} } & {{\mathbf{A}}^{ng} } \\ {{\mathbf{A}}^{gn} } & {{\mathbf{A}}^{gg} + {\mathbf{G}}^{ - 1} - {\mathbf{A}}_{gg}^{ - 1} } \\ \end{array} } \right]$$, and where ${\mathbf{G}} = \left( {\frac{1 - w}{m}{\mathbf{ZZ^{\prime}}} + w{\mathbf{A}}_{gg} } \right)$ is the genomic relationship matrix modified for considering the residual polygenic effects [3, 5].

### A ssPCBLUP model

Due to linkage disequilibrium between SNPs, a small number of PC of the centered genotype matrix ${\mathbf{Z}}$ likely explain most of the genomic variation, while the remaining PC associated with small eigenvalues may reflect noise in the genomic information [14, 25]. Principal components of ${\mathbf{Z}}$ can be obtained by SVD:

$${\mathbf{Z}} = {\mathbf{USV}}',$$

where ${\mathbf{U}}$ and ${\mathbf{V}}$ are unitarian matrices with the left and right singular vectors of ${\mathbf{Z}}$, respectively; and ${\mathbf{S}}$ is a diagonal matrix with non-negative diagonal elements known as singular values (i.e., square roots of the eigenvalues of ${\mathbf{ZZ}}'$ and ${\mathbf{Z}}'{\mathbf{Z}}$). The matrix ${\mathbf{US}}$ is known as the PC score matrix.

Removing the noise can be performed by fitting explicitly only the PC associated with the largest eigenvalues that explain most (e.g., 99%) of the genomic variation, instead of fitting SNP effects, into a ssPCBLUP model as follows [7, 14, 25]:

$${\mathbf{y}} = {\mathbf{Xb}} + \left[ {\begin{array}{*{20}c} {{\mathbf{W}}_{n} } & \mathbf{0} & \mathbf{0} \\ \mathbf{0} & {{\mathbf{W}}_{g} } & {{\mathbf{W}}_{g} \left( {{\mathbf{I}}_{t} \otimes {\mathbf{T}}} \right)} \\ \end{array} } \right]\left[ {\begin{array}{*{20}c} {{\mathbf{u}}_{n} } \\ {{\mathbf{a}}_{g} } \\ {\mathbf{v}} \\ \end{array} } \right] + {\mathbf{e}},$$

where ${\mathbf{v}} = {\mathbf{V}}'{\mathbf{g}}$; and ${\mathbf{T}} = {\mathbf{U}}{\hat{\mathbf{S}}}$ with ${\hat{\mathbf{S}}}$ containing the largest singular values of ${\mathbf{S}}$ corresponding to the largest eigenvalues that explain, e.g. 99%, of the genomic variation.

The linear system of mixed model equations of ssPCBLUP has the same form as the linear system of mixed model equations of ssSNPBLUP (2), except that ${\mathbf{Z}}$ is replaced by ${\mathbf{T}}$ in ${\mathbf{M}}_{z}$, i.e. ${\mathbf{I}}_{t} \otimes {\mathbf{Z}}$ by ${\mathbf{I}}_{t} \otimes {\mathbf{T}}$. It is also worth noting that the number of columns of ${\mathbf{T}}$ (which is the number of PC kept) is smaller than the number of columns of ${\mathbf{Z}}$ (which is the number of SNPs) due to rank reduction.

### Iterative solvers

The linear systems of mixed model equations of ssGBLUP, ssSNPBLUP, and ssPCBLUP have the form:

$${\mathbf{Cx}} = {\mathbf{b}},$$

where ${\mathbf{C}}$ is a SPSD coefficient matrix, ${\mathbf{x}}$ is the vector of solutions, and ${\mathbf{b}}$ is the right-hand-side.

Such linear systems of equations can be solved using direct methods [17]. A bottleneck of most of these methods is that they involve an explicit factorization of ${\mathbf{C}}$. The resulting matrix factor is often dense and might require excessive amounts of memory and computation. Therefore, direct methods are usually too expensive and, in some cases, even impossible for large linear systems. Instead of direct methods, iterative methods, i.e. methods that use successive approximations to obtain more accurate solutions for a linear system at each iteration step, are more attractive. With iterative methods, both memory requirements and computing time can be reduced, especially if ${\mathbf{C}}$ is large and sparse. Within the class of iterative methods, the CG methods are the best choice, especially when ${\mathbf{C}}$ is SPSD [17].

#### Conjugate gradient method and effective spectral condition number

The purpose of CG methods is to construct a sequence, $\left\{ {{\hat{\mathbf{x}}}_{j} } \right\}$, that satisfies ${\hat{\mathbf{x}}}_{j + 1} \in {\hat{\mathbf{x}}}_{0} + {\rm K}_{j} \left( {{\mathbf{C}},{\mathbf{r}}_{0} } \right)$, where ${\hat{\mathbf{x}}}_{0}$ is a vector of starting solutions, ${\mathbf{r}}_{0} = {\mathbf{b}} - {{C\hat{\mathbf{x}}}}_{0}$, and ${\rm K}_{j} \left( {{\mathbf{C}},{\mathbf{r}}_{0} } \right)$ is the Krylov subspace ${\rm K}_{j}$ equal to ${\rm K}_{j} \left( {{\mathbf{C}},{\mathbf{r}}_{0} } \right) = span\left\{ {{\mathbf{r}}_{0} ,{\mathbf{Cr}}_{0} ,{\mathbf{C}}^{2} {\mathbf{r}}_{0} , \ldots ,{\mathbf{C}}^{j - 1} {\mathbf{r}}_{0} } \right\}$. After $j + 1$ iterations, the error is bounded by [17]:

4 $$\left\| {{\mathbf{x}} - \widehat{{\mathbf{x}}}_{{j + 1}} } \right\|_{{\mathbf{A}}} \le 2\left\| {{\mathbf{x}} - \widehat{{\mathbf{x}}}_{0} } \right\|_{{\mathbf{A}}} \left( {\frac{{\sqrt {\kappa \left( {\mathbf{C}} \right)} - 1}}{{\sqrt {\kappa \left( {\mathbf{C}} \right)} + 1}}} \right)^{{j + 1}} ,$$

where ${\mathbf{x}}_{{\mathbf{A}}}$ is the A-norm of ${\mathbf{x}}$, defined as $\sqrt {{\mathbf{x^{\prime}Ax}}}$, $\kappa \left( {\mathbf{C}} \right)$ is the effective spectral condition number of the coefficient matrix ${\mathbf{C}}$ and is defined as $\kappa \left( {\mathbf{C}} \right) = \frac{{\lambda_{max} }}{{\lambda_{ {\min} } }}$ with $\lambda_{ {\min} }$ ($\lambda_{max}$) being the smallest (largest) non-zero eigenvalue of ${\mathbf{C}}$ [26]. The more ${\mathbf{C}}$ is well-conditioned, the smaller is $\kappa \left( {\mathbf{C}} \right)$, the smaller is the error bound, which is expected to result in faster convergence of the CG method [17]. It is worth noting that the convergence of the CG method does not depend only on $\kappa \left( {\mathbf{C}} \right)$, since $\kappa \left( {\mathbf{C}} \right)$ affects only the upper bound of the error (i.e., the worst convergence rate). Indeed, convergence also depends on the clustering of the eigenvalues of the system matrix, on the right-hand-side ${\mathbf{b}}$, and on floating point rounding errors. These factors may lead to different convergence patterns for two different systems of equations with a similar $\kappa \left( {\mathbf{C}} \right)$.

#### Preconditioned conjugate gradient method

To improve the performance of the CG method, the linear system of equations, ${\mathbf{Cx}} = {\mathbf{b}}$, is transformed into an equivalent linear system of equations for which the resulting system matrix, i.e. the preconditioned coefficient matrix, has an effective spectral condition number smaller than $\kappa \left( {\mathbf{C}} \right)$. This can be realized by preconditioning the linear system with a symmetric positive definite matrix ${\mathbf{M}}$, called preconditioner. The resulting preconditioned linear system of equations can be written as follows [17]:

5 $${\mathbf{M}}^{ - 1} {\mathbf{Cx}} = {\mathbf{M}}^{ - 1} {\mathbf{b}} .$$

The preconditioned linear system can be solved with the PCG method using the algorithm given in Table 1. Equation (4) for the error bound of the CG method also applies to the PCG method by replacing $\kappa \left( {\mathbf{C}} \right)$ with $\kappa \left( {{\mathbf{M}}^{ - 1} {\mathbf{C}}} \right)$. Thus, the preconditioner ${\mathbf{M}}$ must be chosen such that $\kappa \left( {{\mathbf{M}}^{ - 1} {\mathbf{C}}} \right) \le \kappa \left( {\mathbf{C}} \right)$. A general rule is that the preconditioner ${\mathbf{M}}$ approximates ${\mathbf{C}}$ to obtain eigenvalues that cluster around 1. The preconditioner ${\mathbf{M}}$ must be also chosen such that inexpensive costs are required for its construction and for the multiplication of its inverse ${\mathbf{M}}^{ - 1}$, with a vector, as this operation is performed at each iteration of the PCG method (Table 1). For linear systems of equations resulting from mixed models, such as models (1) and (3), a preconditioner ${\mathbf{M}}$ equal to the diagonal elements of ${\mathbf{C}}$, i.e. ${\mathbf{M}} = diag\left( {\mathbf{C}} \right)$, is widely used [11, 15, 16, 18, 27]. For multivariate analyses, ${\mathbf{M}}$ is usually defined as a block diagonal matrix [11, 15, 16, 18, 27].

**Table 1:** Algorithm for preconditioned conjugate gradient (PCG) and deflated PCG (DPCG) methods for solving ${\mathbf{x}}$ in the linear system ${\mathbf{Cx}} = {\mathbf{b}}$ using a preconditioner M

1	Select an initial guess ${\mathbf{x}}_{0}$; ${\mathbf{r}}_{\text{init}} = {\mathbf{b}} - {\mathbf{Cx}}_{0}$; ${\mathbf{r}}_{0} = {\mathbf{\psi r}}_{\text{init}}$; ${\mathbf{p}}_{ - 1} = 0$; $\uptau_{ - 1} = 1$
2	for $j = \;0$,…, until convergence
3	${\mathbf{y}}_{j} = {\mathbf{M}}^{ - 1} {\mathbf{r}}_{j}$
4	$\uptau_{j} = {\mathbf{r}}_{j}^{\varvec{'}} {\mathbf{y}}_{j}$
5	$\upbeta_{j} =\uptau_{j} /\uptau_{j - 1}$
6	$\uptau_{j - 1} =\uptau_{j}$
7	${\mathbf{p}}_{j} = {\mathbf{y}}_{j} +\upbeta_{j} {\mathbf{p}}_{j - 1}$
8	${\mathbf{w}}_{j} = {\mathbf{{\varvec{\uppsi}} Cp}}_{j}$
9	$\upalpha_{j} = {\mathbf{r}}_{j}^{ '} {\mathbf{y}}_{j} /{\mathbf{p}}_{j}^{\varvec{'}} {\mathbf{w}}_{j}$
10	${\mathbf{x}}_{j + 1} = {\mathbf{x}}_{j} +\upalpha_{j} {\mathbf{p}}_{j}$
11	${\mathbf{r}}_{j + 1} = {\mathbf{r}}_{j} -\upalpha_{j} {\mathbf{w}}_{j}$
12	end
13	${\mathbf{x}}_{final} = {\varvec{\upupsilon}}$

For PCG: ${\varvec{\uppsi}} = {\mathbf{I}}$, ${\varvec{\upupsilon}} = {\mathbf{x}}_{j + 1}$; for DPCG: ${\varvec{\uppsi}} = {\mathbf{P}} = {\mathbf{I}} - {\mathbf{CZ}}_{d} \left( {{\mathbf{Z}}_{d}^{'} {\mathbf{CZ}}_{d} } \right)^{ - 1} {\mathbf{Z}}_{d}^{'}$, ${\varvec{\upupsilon}} = {\mathbf{Z}}_{d} \left( {{\mathbf{Z}}_{d}^{'} {\mathbf{CZ}}_{d} } \right)^{ - 1} {\mathbf{Z}}_{d}^{'} {\mathbf{b}} + {\mathbf{P^{\prime}x}}_{j + 1}$, and ${\mathbf{Z}}_{d}$ is a deflation-subspace matrix

For the PCG implementation: the equation in line 11 $({\mathbf{r}}_{j + 1} = {\mathbf{r}}_{j} -\upalpha_{j} {\mathbf{w}}_{j} )\varvec{ }$ was replaced by the equation ${\mathbf{r}}_{j + 1} = {\mathbf{b}} - {\mathbf{Ax}}_{j + 1}$ at each 50 iterations [16]

#### Deflated PCG method

The deflated PCG method is a two-level PCG method, which iteratively solves ill-conditioned linear systems of equations, i.e. linear systems of equations with a large effective spectral condition number [19–21]. Large effective spectral condition numbers are obtained when very small, or very large, or both, non-zero eigenvalues are present in the set of eigenvalues, called spectrum, of ${\mathbf{M}}^{ - 1} {\mathbf{C}}$. These very small or very large eigenvalues of a spectrum are called hereafter unfavourable eigenvalues. Deflation is used to annihilate the effect of the most unfavourable eigenvalues of the spectrum of ${\mathbf{M}}^{ - 1} {\mathbf{C}}$ on the convergence of the PCG method by setting these unfavourable eigenvalues to 0 [20]. The deflation is performed by introducing a second-level preconditioner, ${\mathbf{P}}$, also called deflation matrix, into the preconditioned linear system of equations, ${\mathbf{M}}^{ - 1} {\mathbf{Cx}} = {\mathbf{M}}^{ - 1} {\mathbf{b}}$, as follows [19–21]:

$${\mathbf{M}}^{ - 1} {\mathbf{PCx}}_{d} = {\mathbf{M}}^{ - 1} {\mathbf{Pb}},$$

where ${\mathbf{x}}_{d}$ is the vector of deflated solutions and is related to the vector of solutions ${\mathbf{x}}$ of the system of equations ${\mathbf{Cx}} = {\mathbf{b}}$ as ${\mathbf{x}} = {\mathbf{Z}}_{d} {\mathbf{E}}^{ - 1} {\mathbf{Z}}_{d}^{\varvec{'}} {\mathbf{b}} + {\mathbf{P}}'{\mathbf{x}}_{d}$; the deflation matrix ${\mathbf{P}}$ is equal to ${\mathbf{P}} = {\mathbf{I}} - {\mathbf{CZ}}_{d} {\mathbf{E}}^{ - 1} {\mathbf{Z}}_{d}^{\varvec{'}}$; the matrix ${\mathbf{Z}}_{d}$ is the deflation-subspace matrix of rank $k$ that contains $k$ columns, called deflation vectors; and the matrix ${\mathbf{E}} = {\mathbf{Z}}_{d}^{\varvec{'}} {\mathbf{CZ}}_{d}$ is a symmetric positive definite matrix, called Galerkin or coarse matrix [28], which can be easily computed and inverted, or factored if it is too large.

Because Eq. (4) for the error bound of the CG method also applies to the DPCG method by replacing $\kappa \left( {\mathbf{C}} \right)$ with $\kappa \left( {{\mathbf{M}}^{ - 1} {\mathbf{PC}}} \right)$, choosing an adequate combination of ${\mathbf{M}}^{ - 1} {\mathbf{P}}$, i.e. choosing a deflation matrix ${\mathbf{P}}$, and thus a deflation-subspace matrix ${\mathbf{Z}}_{d}$, in combination with ${\mathbf{M}}$, should yield faster convergence. Ideally, matrix ${\mathbf{Z}}_{d}$ should contain the eigenvectors corresponding to the unfavourable eigenvalues of ${\mathbf{M}}^{ - 1} {\mathbf{C}}$ to achieve the fastest convergence [20–22]. However, obtaining and applying such eigenvectors is computationally intensive. Therefore, the deflation vectors of the deflation-subspace matrix ${\mathbf{Z}}_{d}$ should approximate the same space as the span of the unfavourable eigenvectors such that $\kappa \left( {{\mathbf{M}}^{ - 1} {\mathbf{PC}}} \right) \le \kappa \left( {{\mathbf{M}}^{ - 1} {\mathbf{C}}} \right)$. The number ($k$) of deflation vectors should be chosen such that the deflation approach gives good results while the additional computational costs are limited as much as possible. Indeed, the size ($k$) of the Galerkin matrix should be limited so that it can be stored in memory, and the computational costs associated with the multiplication of ${\mathbf{P}} = {\mathbf{I}} - {\mathbf{CZ}}_{d} {\mathbf{E}}^{ - 1} {\mathbf{Z}}_{d}^{\varvec{'}}$ with a vector should be also limited because this operation is performed at each iteration of the DPCG method. For example, if $k = 1$, the computational costs are minimized since ${\mathbf{Z}}_{d}$ is a vector and ${\mathbf{E}}^{ - 1}$ is a scalar. However, in this case, the DPCG method is expected to hardly improve the convergence pattern. Contrariwise, if $k$ is equal to the number of equations of the linear system (i.e., $k$ is large), then ${\mathbf{Z}}_{d}$ and ${\mathbf{E}}^{ - 1}$ are square matrices with the same size as ${\mathbf{C}}$. Furthermore, if ${\mathbf{Z}}_{d}$ is defined as an identity matrix, the DPCG method is equivalent to a direct solver, since ${\mathbf{E}}^{ - 1} = {\mathbf{C}}^{ - 1}$, ${\mathbf{P}} = 0$, and ${\mathbf{x}} = {\mathbf{Z}}_{d} {\mathbf{E}}^{ - 1} {\mathbf{Z}}_{d}^{\varvec{'}} {\mathbf{b}} + {\mathbf{P^{\prime}x}}_{d} = {\mathbf{C}}^{ - 1} {\mathbf{b}}$. In this case, the additional computational costs are equal to the costs of inverting ${\mathbf{C}}$, and the DPCG method will converge in one iteration. The algorithm for the DPCG method is in Table 1.

#### Definition of the deflation-subspace matrix for ssSNPBLUP and ssPCBLUP

The deflation vectors of the deflation-subspace matrix ${\mathbf{Z}}_{d}$ can be defined following several techniques based on, e.g., approximating eigenvectors [29], recycling information of previous Krylov subspaces [21], or subdomain deflation vectors [22]. All these approaches have their own advantages and disadvantages [20, 28]. For example, some advantages of the subdomain deflation approach are that ${\mathbf{Z}}_{d}$ is sparse, and that additional computations for the DPCG method (in comparison to the PCG method) can be implemented efficiently [22]. Due to these advantages and based on preliminary results, the deflation vectors of the deflation-subspace matrix ${\mathbf{Z}}_{d}$ were defined following the subdomain deflation approach in this study [22]. This approach divides the computational domain ${\mathbb{R}}^{n}$ (with ${\mathbf{x}} \in {\mathbb{R}}^{n}$) into $k$ non-overlapping subdomains, with each $i$-th ($i = 1, \ldots , k$) subdomain corresponding to the $i$-th deflation vector. An entry of the deflation vector ${\mathbf{Z}}_{di}$ is equal to 1 if the corresponding entry is included in the $i$-th subdomain; otherwise the entry of ${\mathbf{Z}}_{di}$ is equal to 0. Therefore, each row of ${\mathbf{Z}}_{d}$ contains only one non-zero element. The subdomain deflation approach gives good results if $k$ is large enough [22].

Multiple divisions of the computational domain of ssSNPBLUP (ssPCBLUP) into $k$ non-overlapping subdomains are possible to define the required deflation-subspace matrix ${\mathbf{Z}}_{d}$. The optimal division depends on the properties of the linear system of equations. For example, Vuik et al. [20] defined the subdomains based on the properties of the eigenvectors associated with the smallest eigenvalues of ${\mathbf{M}}^{ - 1} {\mathbf{C}}$ for a class of layered problems with extreme contrasts in ${\mathbf{C}}$. This approach could not be extended to ssSNPBLUP because we were not able to identify which model terms were associated with the most unfavourable eigenvalues of ${\mathbf{M}}^{ - 1} {\mathbf{C}}$ (results not shown). However, based on the observation that fitting SNP effects explicitly led to an increase of the largest eigenvalues of ${\mathbf{M}}^{ - 1} {\mathbf{C}}$ for ssSNPBLUP, in comparison to ssGBLUP (see sections Results and Discussion), we hypothesized that grouping SNP effects in subdomains could enable the DPCG method to annihilate the effects of the most unfavourable eigenvalues. Thus, we divided the ssSNPBLUP domain per trait and then within trait as follows: (1) all effects associated with the same trait and other than the SNP effects were included in a separate subdomain; and (2) each set of $l$ randomly chosen SNP effects associated with the same trait were included in a separate subdomain. Following this division, the number of subdomains $k$, and therefore the rank of ${\mathbf{Z}}_{d}$ and the size of the Galerkin matrix ${\mathbf{E}}$, is equal to $k = \left( {1 + \frac{{n_{snp} }}{l}} \right)*t$, where $n_{snp}$ is the number of SNPs, and $\frac{{n_{snp} }}{l}$ is equal to the smallest integer greater than or equal to $\frac{{n_{snp} }}{l}$. It is also worth noting that the proposed division of the ssSNPBLUP domain with $l = 1$ SNP effect per subdomain leads to a system matrix ${\mathbf{M}}^{ - 1} {\mathbf{PC}}$ with zero entries for all equations associated with the SNP effects (see Additional file 1). Because the behaviour of the PCG method applied to ssPCBLUP was similar to that of the PCG method applied to ssSNPBLUP (see the Results section), we divided the ssPCBLUP domain with the same approach as for the ssSNPBLUP domain, except that each set of PC effects included $l$ consecutive PC effects, which were sorted following a descending order of their associated eigenvalues. In this study, four different deflation-subspace matrices for ssSNPBLUP (ssPCBLUP) were defined by means of sets of $l =$ 1, 5, 50, and 200 SNP (PC) effects.

#### Termination criteria

Because the PCG and DPCG methods are iterative methods, termination criteria must be defined to determine when the methods have reached convergence. In this study, $\frac{{{\mathbf{r}}_{j + 1} }}{{\mathbf{b}}} \le \delta$ and $\frac{{\varvec{r}_{dj + 1} }}{{\mathbf{b}}} \le \delta$ were used as termination criteria for the PCG and DPCG methods, respectively, with $\left\| \cdot \right\|$ being the 2-norm, and $r_{dj + 1}$ being the residual of the deflated system after $j + 1$ iterations. It has been shown that the residual of the PCG method is the same as the residual of the DPCG method [20]. Therefore, the two termination criteria are the same.

### Data and models

Two datasets, a reduced dataset and a field dataset, were provided by CRV BV (The Netherlands). To achieve the first aim of this study, the reduced dataset was used to compare properties of the system matrices (${\mathbf{M}}^{ - 1} {\mathbf{C}}$ or ${\mathbf{M}}^{ - 1} {\mathbf{PC}}$) of ssSNPBLUP and of ssGBLUP, and to relate this to observed convergence patterns. To achieve the second aim of this study, the field dataset was used to test the feasibility of the DPCG method for solving the linear system of equations associated with ssSNPBLUP and ssPCBLUP applied to large multi-trait datasets.

The reduced dataset and associated variance components were extracted from the Dutch single-step genomic evaluation from August 2017 for ovum pick-up (OPU) and embryo transfer of Holstein dairy cattle. After extraction of the OPU sessions, the data file included 61,592 OPU sessions from 4109 animals, and the pedigree included 37,021 animals. The genotypes of 6169 animals without phenotype were available. Bulls were genotyped using the Illumina 50 K SNP chip. Cows were genotyped using the Illumina 3 K chip and were imputed to 50 K density using a combination of the Phasebook software [30] and Beagle [31]. Because some currently used (own) libraries cannot handle sparse matrices with more than 2^31^–1 elements, and also to keep the system of equations at a reasonable size for subsequent analyses (e.g., for the computation of all eigenvalues), genotypes included 9994 segregating SNPs with a minor allele frequency higher than or equal to 0.01 and randomly sampled from (imputed) 50 K SNP genotypes. The univariate mixed model included random effects (additive genetic, permanent environmental, and residual), fixed co-variables (heterosis and recombination), and fixed cross-classified effects (herd-year, year-month, parity, age (in months), technician, assistant, interval, gestation, session, and protocol) [32].

The field dataset and associated variance components were from the 4-trait routine genetic evaluation of August 2017 for temperament and milking speed of dairy cattle for the Netherlands and the Flemish region in Belgium [33, 34]. Performance in both countries were considered as different traits, with genetic correlations between Flemish and Dutch traits higher than 0.85. The data file included 3882,772 records with a single record per animal. The pedigree included 6130,519 animals. The genotypes of 15,205 animals without phenotype and of 75,758 animals with phenotype were available. Animals were genotyped in the same manner as described above. After removing non-segregating SNPs and SNPs with a minor allele frequency lower than 0.01, genotypes included 37,995 segregating SNPs. The four-trait mixed model included random effects (additive genetic and residual), fixed co-variables (heterosis and recombination), and fixed cross-classified (herd × year × season at classification, age at classification, lactation stage at classification, milk yield and month of calving). More details on this genetic evaluation can be found in [33, 34].

For both datasets, genetic groups were removed from the pedigrees (for simplicity), the proportion $w$ for residual polygenic effects was assumed to be equal to 0.05, and the centered genotype matrices ${\mathbf{Z}}$ for ssSNPBLUP and the matrices ${\mathbf{G}}^{ - 1} - {\mathbf{A}}_{gg}^{ - 1}$ for ssGBLUP were computed with the software calc_grm [35]. For the field dataset only, a matrix ${\mathbf{T}}$ that contained PC kept for ssPCBLUP was also computed using the software calc_grm. The number of PC kept was equal to the number of the largest eigenvalues that together explain 99% of the genomic variation.

### Statistical analyses

#### Computation of eigenvalues and effective spectral condition numbers

Properties of the system matrices and convergence patterns of ssSNPBLUP and ssPCBLUP were compared to those of ssGBLUP. For the reduced dataset, the spectrum of the preconditioned coefficient matrix, ${\mathbf{M}}^{ - 1} {\mathbf{C}}$, of both ssGBLUP and ssSNPBLUP, and of the preconditioned deflated coefficient matrix, ${\mathbf{M}}^{ - 1} {\mathbf{PC}}$, of ssSNPBLUP with different deflation-subspace matrices, were computed using Intel(R) Math Kernel Library (MKL) 11.3.2 subroutines. For the field dataset, due to the large number of equations (> 10^7^), the smallest and largest positive eigenvalues of the different system matrices of ssSNPBLUP, and ssPCBLUP were approximated using the Lanczos method based on information obtained from the (D)PCG method [36, 37]. Performed with the (D)PCG method, the Lanczos method approximates the smallest and largest eigenvalues that influence the convergence. Because the null space of a system matrix never enters the iteration of the (D)PCG method, the corresponding zero eigenvalues do not influence the convergence and therefore are not approximated with the Lanczos method [20, 22, 38, 39]. It is also worth noting that the precision of the approximations of the eigenvalues may vary between analyses, which can partly explain the fact that the number of iterations to reach convergence may not be completely related with the associated effective spectral condition number. The different $\kappa \left( {{\mathbf{M}}^{ - 1} {\mathbf{C}}} \right)$ and $\kappa \left( {{\mathbf{M}}^{ - 1} {\mathbf{PC}}} \right)$ were thereafter computed to compare the different systems of equations.

#### Solving ssSNPBLUP, ssPCBLUP, and ssGBLUP

Linear systems of equations of ssSNPBLUP, of ssPCBLUP, and of ssGBLUP, were solved by using the PCG method. Systems of ssSNPBLUP were also solved by using the DPCG method with sets of 1, 5, 50, and 200 randomly chosen SNP effects per subdomain for the reduced dataset, and with sets of 5, 50, and 200 randomly chosen SNP effects per subdomain for the field dataset. The set of 1 SNP effect per subdomain was not used for the field dataset due to a Galerkin matrix of size 155,980, which was considered as too large for inversion. Similarly, systems of ssPCPBLUP were solved by using the DPCG method with sets of 1, 5, 50, and 200 consecutive PC effects per subdomain for the field dataset. For both the PCG and DPCG methods, the iterative process was run for a maximum of 10,000 iterations, or until termination criteria reached $\delta = 10^{ - 6}$. In addition, for both the PCG and DPCG methods, the preconditioner ${\mathbf{M}}$ was equal to:

$${\mathbf{M}} = \left[ {\begin{array}{*{20}c} {{\mathbf{M}}_{ff} } & \mathbf{0} \\ \mathbf{0} & {{\mathbf{M}}_{rr} } \\ \end{array} } \right] = \left[ {\begin{array}{*{20}c} {diag\left( {{\mathbf{C}}_{ff} } \right)} & \mathbf{0} \\ \mathbf{0} & {block - diag\left( {{\mathbf{C}}_{rr} } \right)} \\ \end{array} } \right],$$

where the subscripts $f$ and $r$ refer to the equations associated with fixed and random effects, respectively, and $block - diag\left( {{\mathbf{C}}_{rr} } \right)$ is a block-diagonal matrix with blocks corresponding to equations for different traits within a level (e.g., an animal). For the field dataset, diagonal elements of ${\mathbf{Q}} = {\mathbf{A}}^{gn} \left( {{\mathbf{A}}^{nn} } \right)^{ - 1} {\mathbf{A}}^{ng}$ were approximated using a Monte Carlo method [40, 41].

Linear systems of equations of ssSNPBLUP, of ssPCBLUP, and of ssGBLUP were solved by using a Fortran 2003 program exploiting BLAS and sparse BLAS routines and the parallel direct sparse solver PARDISO, all from the multi-threaded Intel Math Kernel Library 11.3.2, and OpenMP parallel computing. For the reduced dataset, the coefficient matrix ${\mathbf{C}}$ was held in memory using a compressed sparse row format, and the multiplication of ${\mathbf{P}}$ by a vector ${\mathbf{v}}$ required by the DPCG method, was performed as ${\mathbf{Pv}} = {\mathbf{v}} - \left[ {{\varvec{\Gamma}}\left[ {{\mathbf{E}}^{ - 1} \left[ {{\mathbf{Z}}_{d}^{\varvec{'}} {\mathbf{v}}} \right]} \right]} \right]$, where the brackets $\left[ \cdot \right]$ indicate the order of the matrix–vector operations. The matrices ${\mathbf{E}}^{ - 1}$ and ${\varvec{\Gamma}} = {\mathbf{CZ}}_{d}$ were readily computed from the coefficient matrix ${\mathbf{C}}$ held in memory. These matrices were computed before starting the iterative process and held in memory. The number of OpenMP threads was limited to 3 for the reduced dataset.

For the field dataset, the coefficient matrix ${\mathbf{C}}$ was reconstructed using a matrix-free approach when required for its multiplication by a vector. The matrix ${\mathbf{E}}^{ - 1}$ was held in memory. The computation of ${\mathbf{E}}$ was not as straightforward as for the reduced dataset because the coefficient matrix ${\mathbf{C}}$ was not held in memory. Therefore, the matrix ${\mathbf{E}}$ was computed following a suboptimal 4-step approach. The first step consisted of sequentially pre- and post-multiplying the coefficient matrix ${\mathbf{C}}$ by the first $t$ deflation vectors of the deflation-subspace matrix ${\mathbf{Z}}_{d}$, i.e., the deflation vectors corresponding to the subdomains that included all effects associated with a same trait and other than the SNP effects. The second step consisted of computing each vector ${\mathbf{Z}}_{d,sub}^{'} {\mathbf{M}}_{z}^{\varvec{'}} {\mathbf{W}}_{g}^{'} {\mathbf{R}}_{g}^{ - 1} {\mathbf{W}}_{g} {\mathbf{M}}_{z} {\mathbf{z}}_{d,o}$ sequentially with ${\mathbf{Z}}_{d,sub}$ being a sub-matrix of ${\mathbf{Z}}_{d}$ with the row entries corresponding to the SNP effects, and ${\mathbf{z}}_{d,o}$ being the $o$-th vector of ${\mathbf{Z}}_{d,sub}$ with $o = t + 1, \ldots ,k$. The third and fourth steps consisted of computing ${\mathbf{Z}}_{d,sub}^{'} \left( {{\mathbf{G}}_{0}^{ - 1} \otimes {\mathbf{Z^{\prime}QZ}}} \right){\mathbf{Z}}_{d,sub}$ and ${\mathbf{Z}}_{d,sub}^{'} \left( {\frac{m}{1 - w}{\mathbf{G}}_{0}^{ - 1} \otimes {\mathbf{I}}} \right){\mathbf{Z}}_{d,sub}$, respectively, with the matrices ${\mathbf{Z^{\prime}QZ}}$ and $\frac{m}{1 - w}{\mathbf{G}}_{0}^{ - 1}$ computed explicitly beforehand. Furthermore, because the matrix ${\varvec{\Gamma}} = {\mathbf{CZ}}_{\varvec{d}}$ was too large to be held in memory for the field dataset, the multiplication of ${\mathbf{P}}$ by a vector ${\mathbf{v}}$ required by the DPCG method, was performed as ${\mathbf{Pv}} = {\mathbf{v}} - \left[ {{\mathbf{C}}\left[ {{\mathbf{Z}}_{d} \left[ {{\mathbf{E}}^{ - 1} \left[ {{\mathbf{Z}}_{d}^{ '} {\mathbf{v}}} \right]} \right]} \right]} \right]$. Due to this latter implementation of the multiplication of ${\mathbf{P}}$ by ${\mathbf{v}}$, each iteration of the DPCG method requires two matrix (${\mathbf{C}}$)-vector products, instead of one matrix–vector product for the PCG method. The number of OpenMP threads was limited to 5 for the field dataset.

All real vectors and matrices were stored using double precision real numbers, except for the preconditioner, which was stored using single precision real numbers. All computations were performed on a computer with 528 GB and running RedHat 7.4 (x86_64) with an Intel Xeon E5-2667 (3.20 GHz) central processing unit processor with 32 cores. Main random access memory (RAM) and time requirements are reported for the field dataset. All reported times are indicative, because they may have been influenced by other jobs running simultaneously on the computer.

## Results

### Comparison of estimates of different single-step BLUP

Estimates for all fixed effects, additive genetic effects, and other possible random effects, of ssGBLUP solved with the PCG method, of ssSNPBLUP solved with the PCG and DPCG methods, and of ssPCBLUP solved with the PCG and DPCG methods, were (almost) the same after convergence was reached. For example, Pearson correlations of all estimates of ssGBLUP solved with the PCG method and the corresponding estimates of ssSNPBLUP solved with the DPCG method using 5 SNP effects per subdomain were higher than 0.999 for both the reduced and field datasets (Table 2) and (see Additional file 2: Figure S1). Regression of estimates of ssGBLUP on estimates of ssSNPBLUP solved with the DPCG method using 5 SNP effects per subdomain led to regression coefficients close to 1 and intercepts close to 0 (Table 2). Similar results were obtained for ssPCBLUP solved with the DPCG method using 1 PC effect per subdomain for the field dataset (Table 2) and (see Additional file 2: Figure S2).

**Table 2:** Comparison of estimates obtained with different models against estimates obtained with ssGBLUP using the PCG method

Dataset	Model^a^	Pearson correlation	Intercept^b^	Regression coefficient^b^
Reduced dataset	ssSNPBLUP	> 0.999	0.045	0.998
Field dataset	ssSNPBLUP	0.999	− 0.001	0.997
	ssPCBLUP	0.999	− 0.001	0.998

^a^ssSNPBLUP was solved with the DPCG method with five SNP effects per subdomain; ssPCBLUP was solved with one PC effect per subdomain

^b^Results from the regression of estimates of ssGBLUP on estimates of ssSNPBLUP or of ssPCBLUP

### Reduced dataset

**Fig. 1:**
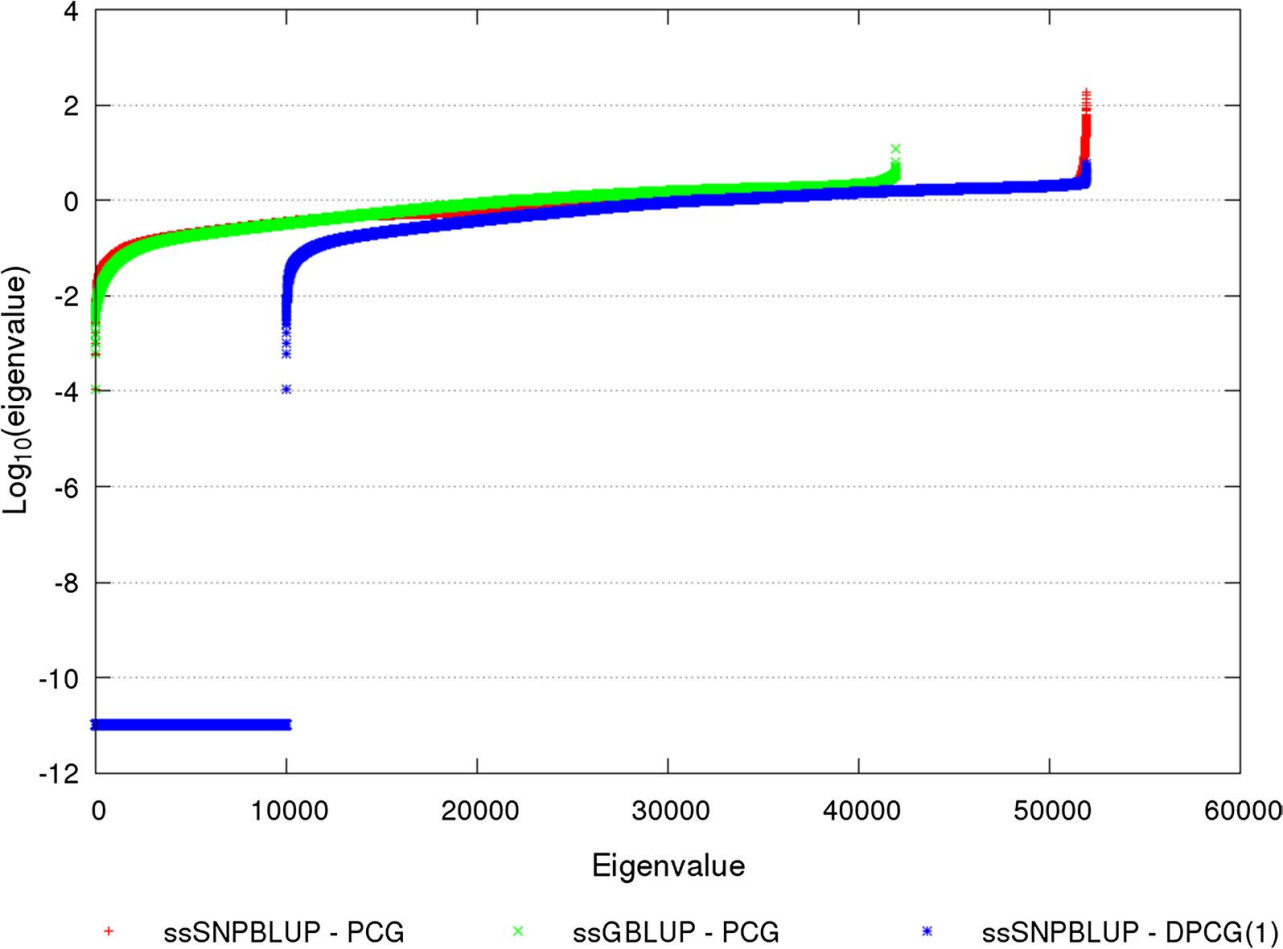
Eigenvalues of different preconditioned (deflated) coefficient matrices for the reduced dataset. Eigenvalues of the preconditioned coefficient matrices of ssGBLUP and of ssSNPBLUP, and of the preconditioned deflated coefficient matrix of ssSNPBLUP with one SNP effect per subdomain are depicted on a logarithm scale. All eigenvalues less than 10^−11^ were set to 10^−11^. Eigenvalues are sorted in ascending order

For the reduced dataset, the number of equations was equal to 41,949 for ssGBLUP and to 51,943 for ssSNPBLUP. Figure 1 shows the spectrum of ${\mathbf{M}}^{ - 1} {\mathbf{C}}$ of ssGBLUP and of ssSNPBLUP, and the spectrum of ${\mathbf{M}}^{ - 1} {\mathbf{PC}}$ of ssSNPBLUP with 1 SNP effect per subdomain. All eigenvalues less than 10^−11^ were assumed to be non-zero due to, for example, rounding errors, and therefore they were set to zero for subsequent analyses. Similar patterns for the different spectra were observed. The smallest non-zero eigenvalues of the different ${\mathbf{M}}^{ - 1} {\mathbf{C}}$ and ${\mathbf{M}}^{ - 1} {\mathbf{PC}}$ that influenced convergence, were equal to 1.1 × 10^−4^, regardless of the model or the definition of subdomains. The largest eigenvalue of ${\mathbf{M}}^{ - 1} {\mathbf{C}}$ was equal to 12 for ssGBLUP, and 181 for ssSNPBLUP (Table 3). When deflation was applied, the largest eigenvalue of ${\mathbf{M}}^{ - 1} {\mathbf{PC}}$ varied from 6 with 1 or 5 SNP effects per subdomain to 99 with 200 SNP effects per subdomain. Deflation of the largest eigenvalues of ${\mathbf{M}}^{ - 1} {\mathbf{C}}$ of ssSNPBLUP can be also observed in Fig. 1. After deflation, the effective spectral condition number of ssSNPBLUP decreased from 1.7 × 10^6^ to between 5.9 × 10^4^ with 1 SNP effect per subdomain and 9.3 × 10^5^ with 200 SNP effects per subdomain.

**Table 3:** Characteristics of preconditioned (deflated) coefficient matrices, and of PCG and DPCG methods for the reduced dataset

Model	Method^a^	Smallest eigenvalue	Largest eigenvalue	Effective condition number	Number of iterations	Total time^b^	Time/iteration^c^
ssGBLUP	PCG	1.1 × 10^−4^	11.9	1.1 × 10^5^	270	11.3	0.05
ssSNPBLUP	PCG	1.1 × 10^−4^	181.0	1.7 × 10^6^	1475	688.2	0.46
	DPCG (200)	1.1 × 10^−4^	99.4	9.3 × 10^5^	1221	570.5	0.47
	DPCG (50)	1.1 × 10^−4^	40.5	3.8 × 10^5^	890	437.7	0.49
	DPCG (5)	1.1 × 10^−4^	6.4	6.0 × 10^4^	331	170.1	0.49
	DPCG (1)	1.1 × 10^−4^	6.0	5.9 × 10^4^	270	189.6	0.66

^a^Number of SNP effects per subdomain is within brackets

^b^Wall clock time (s) for the iterative process

^c^Average wall clock time (s) per iteration. Iterations computing the residual from the coefficient matrix for the PCG method were removed before averaging

**Fig. 2:**
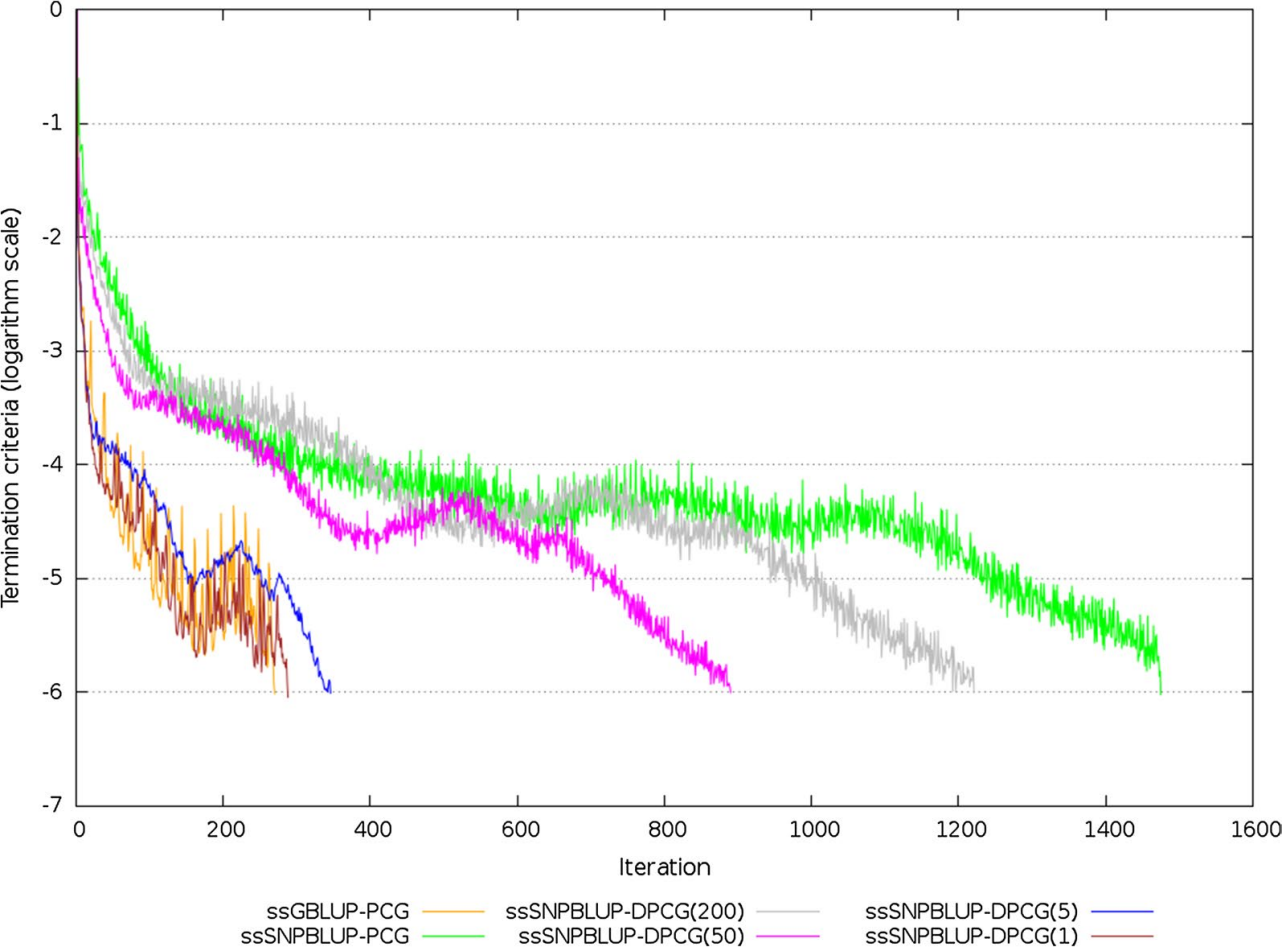
Termination criteria for the reduced dataset for ssGBLUP and ssSNPBLUP using the PCG method and for ssSNPBLUP using the DPCG method. Number of SNP effects per subdomain is within brackets

Both the PCG and DPCG methods reached the termination criteria within 10,000 iterations, and converged to the same solutions for all linear systems of ssGBLUP and ssSNPBLUP. When the PCG method was used, the number of iterations to reach convergence was more than 5 times larger for ssSNPBLUP compared to ssGBLUP (Table 3; Fig. 2). However, when the DPCG method with 1 SNP effect per subdomain was used, the number of iterations decreased by a factor 5, and was similar to the number of iterations needed for ssGBLUP. Five, 50 and 200 SNP effects per subdomain also led to a decrease of the number of iterations by a factor 4.3, 1.7 and 1.3, respectively (Table 3). Figure 2 depicts termination criteria by iteration for the PCG and DPCG methods. A flat pattern is observed for the PCG method applied to ssSNPBLUP. The DPCG method allowed removing this flat pattern such that a pattern similar to that of ssGBLUP was observed.

Regarding the wall clock time per iteration, when the PCG method was applied, about 0.05 s and 0.46 s were needed for ssGBLUP and ssSNPBLUP, respectively. The wall clock time for the iterative process to reach convergence, i.e. excluding the time needed for I/O operations and computations of different matrices (e.g., ${\mathbf{Z}}$, ${\mathbf{G}}^{ - 1} - {\mathbf{A}}_{gg}^{ - 1}$, ${\mathbf{E}}^{ - 1}$, ${\mathbf{M}}^{ - 1}$), was about 11 s for ssGBLUP, and about 688 s for ssSNPBLUP solved with the PCG method. When the DPCG method was applied, the time per iteration for ssSNPBLUP slightly increased due to additional computations involving the deflation matrix ${\mathbf{P}}$. However, the total time for the iterative process decreased to a minimum value of 170 s with 5 SNP effects per subdomain (Table 3).

### Field dataset

**Fig. 3:**
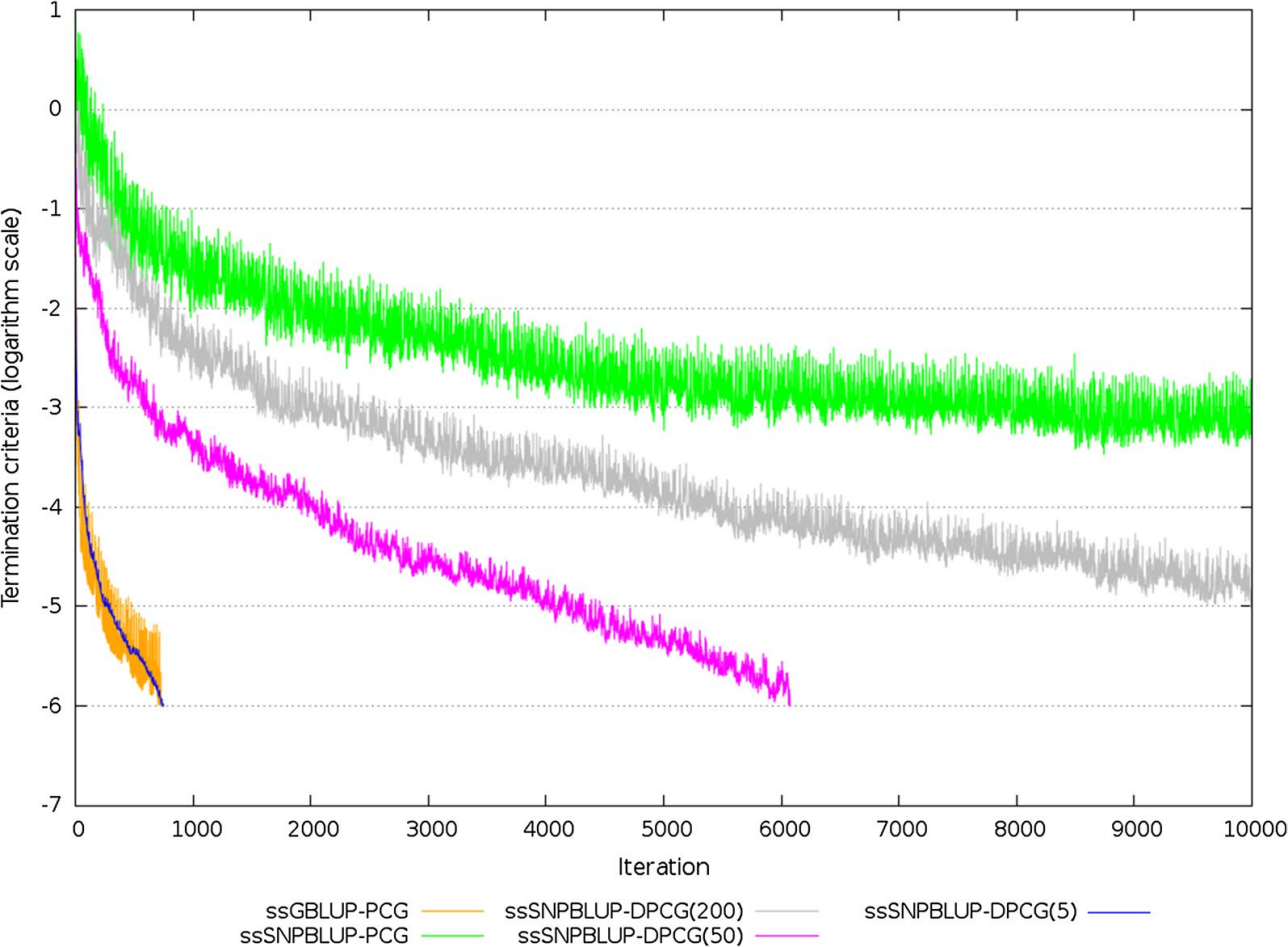
Termination criteria for the field dataset for ssGBLUP and ssSNPBLUP using the PCG method and for ssSNPBLUP using the DPCG method. Number of SNP effects per subdomain is within brackets

**Fig. 4:**
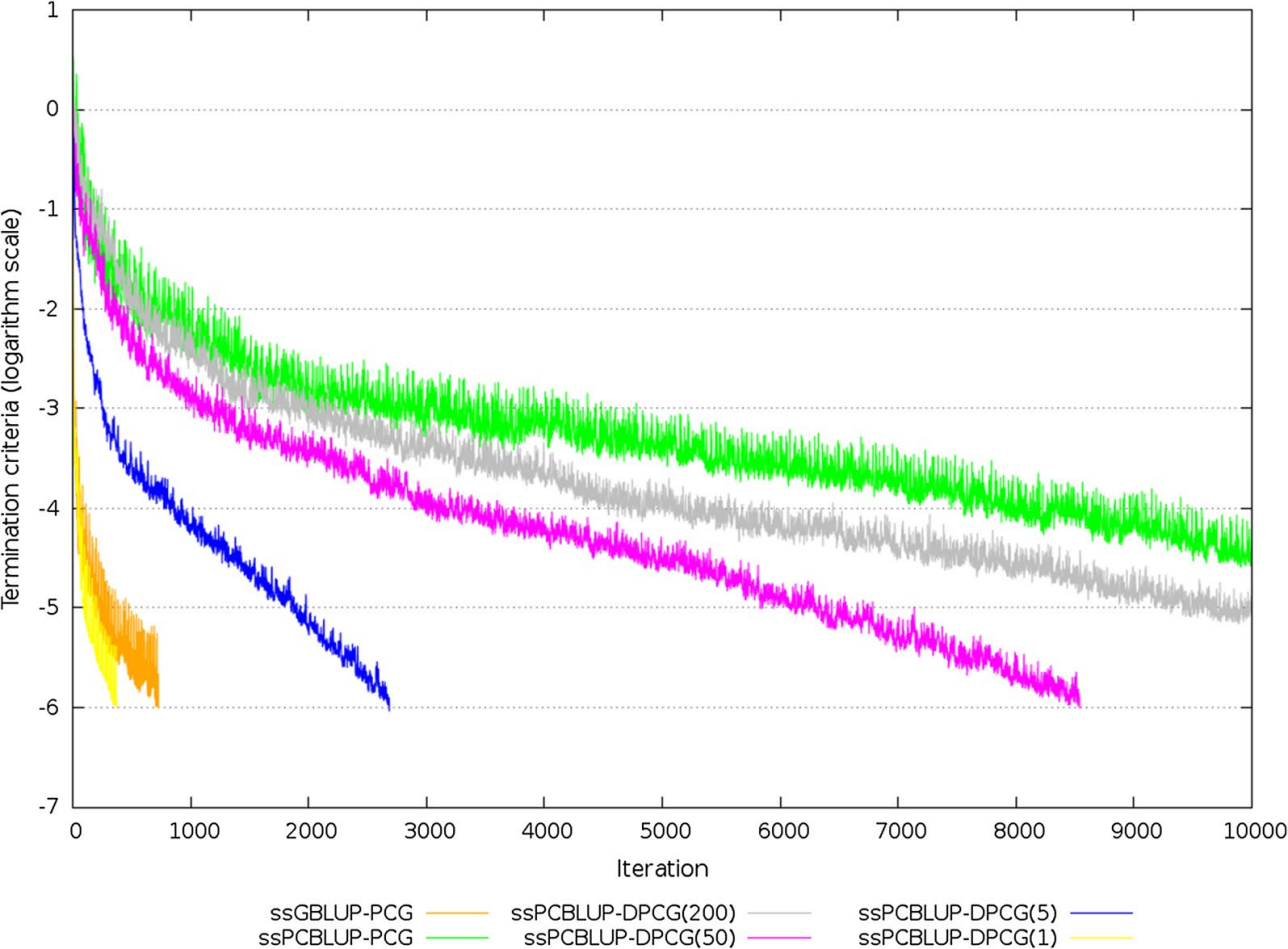
Termination criteria for the field dataset for ssGBLUP and ssPCBLUP using the PCG method and for ssPCBLUP using the DPCG method. The number of principal components retained was equal to 13,803. Number of PC effects per subdomain is within brackets

For the field dataset, the number of equations was larger than 25.8 × 10^6^ for all systems of equations. In total, 13,803 largest eigenvalues of ${\mathbf{Z}}$ explained 99% of the genomic variation. The smallest and largest non-zero eigenvalues that influenced the convergence were estimated using the Lanczos algorithm based on information obtained from the (D)PCG method. The smallest positive eigenvalues of the different ${\mathbf{M}}^{ - 1} {\mathbf{C}}$ and ${\mathbf{M}}^{ - 1} {\mathbf{PC}}$ were estimated between 7.7 × 10^−6^ and 9.6 × 10^−4^. The estimated largest eigenvalue of ${\mathbf{M}}^{ - 1} {\mathbf{C}}$ was equal to 5 for ssGBLUP, to 1752 for ssSNPBLUP, and to 220 for ssPCBLUP (Table 4). When deflation was applied, the estimated largest eigenvalue of ${\mathbf{M}}^{ - 1} {\mathbf{PC}}$ of ssSNPBLUP varied from 5 with 5 SNP effects per subdomain to 193 with 200 SNP effects per subdomain. Similar largest eigenvalues were obtained for ssPCBLUP when deflation was applied. After deflation, the effective spectral condition number of ssSNPBLUP decreased from 4.7 × 10^7^ to between 1.7 × 10^5^ with 5 SNP effects per subdomain and 1.6 × 10^7^ with 200 SNP effects per subdomain (Table 4). For ssPCBLUP, the effective spectral condition number decreased from 1.8 × 10^7^ to between 4.9 × 10^4^ with 1 PC effect per subdomain and 1.4 × 10^7^ with 200 PC effects per subdomain (Table 4). Only the PCG method for ssGBLUP, the DPCG method for ssSNPBLUP with 5 and 50 SNP effects per subdomain, and the DPCG method for ssPCBLUP with 1 to 50 PC effects per subdomain converged within 10,000 iterations (Table 4; Figs. 3, 4). The other (D)PCG methods for ssSNPBLUP and for ssPCBLUP were stopped after 10,000 iterations. For ssSNPBLUP, the termination criteria at the 10,000-th iteration was equal to 8.0 × 10^−4^ for the PCG method, and to 1.3 × 10^−5^ for the DPCG method with 200 SNP effects per subdomain. For ssPCBLUP, the termination criteria at the 10,000-th iteration was equal to 3.9 × 10^−5^ for the PCG method, and to 9.4 × 10^−6^ for the DPCG method with 200 PC effects per subdomain (Table 4; Figs. 3, 4).

**Table 4:** Characteristics of preconditioned (deflated) coefficient matrices, and of PCG and DPCG methods for the field dataset

Model	Method^a^	Smallest eigenvalue	Largest eigenvalue	Effective condition number	Number of iterations^b^	Total time^c^	Time/iteration^d^
ssGBLUP	PCG	2.3 × 10^−5^	5.1	2.2 × 10^5^	729	3993	5.3 (0.4)
ssSNPBLUP	PCG	3.7 × 10^−5^	1751.9	4.7 × 10^7^	10,000	52,683	4.4 (0.4)
	DPCG (200)	1.2 × 10^−5^	193.1	1.6 × 10^7^	10,000	92,171	9.2 (1.4)
	DPCG (50)	8.7 × 10^−6^	29.9	3.4 × 10^6^	6074	52,503	8.6 (2.4)
	DPCG (5)	2.9 × 10^−5^	4.8	1.7 × 10^5^	748	7735	8.7 (0.3)
ssPCBLUP^e^	PCG	1.2 × 10^−5^	220.0	1.8 × 10^7^	10,000	30,198	2.9 (0.2)
	DPCG (200)	8.3 × 10^−6^	113.3	1.4 × 10^7^	10,000	58,280	5.8 (0.7)
	DPCG (50)	7.7 × 10^−6^	46.0	6.0 × 10^6^	8541	55,388	6.5 (0.5)
	DPCG (5)	8.0 × 10^−6^	5.1	6.4 × 10^5^	2686	15,063	5.6 (0.2)
	DPCG (1)	9.6 × 10^−4^	4.8	4.9 × 10^4^	375	2402	6.3 (0.2)

^a^Number of SNP effects per subdomain is within brackets

^b^A number of iterations equal to 10,000 means that the method failed to converge within 10,000 iterations

^c^Wall clock time (s) for the iterative process

^d^Average wall clock time (s) (SD within brackets) per iteration. Iterations computing the residual from the coefficient matrix for the PCG method were removed before averaging

^e^The number of principal components retained was equal to 13,803

Regarding the wall clock time per iteration, when the PCG method was applied, about 5 s were needed for ssGBLUP and ssSNPBLUP, whereas about 3 s were needed for ssPCBLUP. When the DPCG method was applied for ssSNPBLUP (ssPCBLUP), the time per iteration increased to about 9 (6) s, regardless of the number of SNP (PC) effects per subdomain was used. The wall clock time for the iterative process to reach convergence, that is excluding the time needed for I/O operations and computations of different matrices (e.g., ${\mathbf{Z}}$, ${\mathbf{T}}$, ${\mathbf{E}}^{ - 1}$, ${\mathbf{M}}^{ - 1}$) was equal to 7735 s for ssSNPBLUP solved by the DPCG method with 5 SNP effects per subdomain, and to 2402 s for ssPCBLUP solved by the DPCG method with 1 PC effect per subdomain (Table 4). For comparison, the wall clock time for 10,000 iterations of the PCG method (i.e., the iterative process that never reached convergence) was equal to 52,683 s for ssSNPBLUP and to 30,198 s for ssPCBLUP.

Regarding RAM and time requirements for the field dataset during the solving process, the peak RAM was about 70 GB for ssGBLUP, about 34 GB for ssSNPBLUP, and about 17 GB for ssPCBLUP, when the PCG method was used (Table 5). The peak RAM increased to a maximum of 51 GB when the DPCG method was used (Table 5). Most RAM was used for storing dense matrices (e.g., ${\mathbf{G}}^{ - 1} - {\mathbf{A}}_{gg}^{ - 1}$, ${\mathbf{T}}$, ${\mathbf{Z}}$, and some ${\mathbf{E}}^{ - 1}$; Table 5). The time to compute the Galerkin matrix and its inverse (${\mathbf{E}}^{ - 1}$) using a suboptimal approach varied from 430 to 9630 s (Table 5). It is worth noting that the wall clock time required by calc_grm for the different computations and I/O operations, was equal to 17,071 s (using 7 threads) for the matrix ${\mathbf{G}}^{ - 1} - {\mathbf{A}}_{gg}^{ - 1}$, and 15,663 s (using 10 threads) for the matrix ${\mathbf{T}}$. Times are indicative, because different jobs were run at the same time and different approaches were used for I/O operations.

**Table 5:** Computational costs for different matrices and for the software used for the field dataset

Model	Method^a^	Galerkin matrix (E^−1^)			Dense matrix^d^	${\mathbf{G}}^{ - 1} - {\mathbf{A}}_{{\varvec{gg}}}^{ - 1}$	Software peak memory^e^
		Size^b^	GB	Time^c^ (s)	GB	GB	GB
ssGBLUP	PCG	–	–	–	–	63.1	70.2
ssSNPBLUP	PCG	–	–	–	26.4	–	34.0
	DPCG (200)	764	0.004	2199	26.4	–	43.8
	DPCG (50)	3044	0.071	2959	26.4	–	43.9
	DPCG (5)	30,400	7.1	9131	26.4	–	51.0
ssPCBLUP	PCG	–	–	–	9.6	–	16.6
	DPCG (200)	284	< 0.001	430	9.6	–	16.8
	DPCG (50)	1112	0.009	663	9.6	–	16.8
	DPCG(5)	11,048	0.9	1965	9.6	–	17.7
	DPCG (1)	55,216	23.3	9630	9.6	–	40.6

^a^Number of SNP (PC) effects per subdomain is within brackets

^b^The size of the Galerkin matrix is equal to the rank of the deflation-subspace matrix

^c^Wall clock time required for the computation of the Galerkin matrix following a naive implementation, and computation of its inverse

^d^The dense matrix is the centered genotype matrix ${\mathbf{Z}}$ for ssSNPBLUP and the matrix with principal components ${\mathbf{T}}$ for ssPCBLUP

^e^The software peak memory is defined as the peak resident set size (VmHWM) obtained from the Linux/proc virtual file system

## Discussion

The first aim of this study was to compare the properties of the system matrices of ssSNPBLUP and ssGBLUP, and to relate this to observed convergence patterns of ssSNPBLUP obtained with the PCG method. The second aim of this study was to implement and test the feasibility of a DPCG method for efficient solving of ssSNPBLUP and of ssPCBLUP. These aims are important initial steps in the development of an efficient large-scale genomic evaluation system that can handle hundreds of thousands of animals and probably will use ~ 50 K SNPs. As far as we know, this is the first time that effective spectral condition numbers were computed and that the DPCG method was used in the context of quantitative genetics and of linear mixed models. The obtained results showed that larger eigenvalues were obtained for the preconditioned coefficient matrix ${\mathbf{M}}^{ - 1} {\mathbf{C}}$ when SNP effects were fitted explicitly in the model, in comparison to ssGBLUP. Since the smallest eigenvalues of ${\mathbf{M}}^{ - 1} {\mathbf{C}}$ of ssSNPBLUP were similar to those of ${\mathbf{M}}^{ - 1} {\mathbf{C}}$ of ssGBLUP, larger effective spectral condition numbers were obtained for ssSNPBLUP solved by the PCG method, in comparison to ssGBLUP. Thus, this increase of the largest eigenvalues can be associated with the slow convergence of the PCG method applied to ssSNPBLUP. Deflating the largest eigenvalues allowed faster convergence of the DPCG method applied to ssSNPBLUP. Interestingly, similar, or slightly improved, condition numbers and convergence patterns were obtained for ssPCBLUP, while noise in the genomic information was removed by fitting explicitly PC effects instead of SNP effects. These results can be explained by the fact that the eigenvalues associated with the matrix ${\mathbf{T}}$ and the corresponding eigenvalues associated with the centered genotype matrix ${\mathbf{Z}}$ are the same, and suggest that convergence issues with the PCG method are not (only) due to noise in the genomic information.

The proposed definition of the deflation(-subspace) matrix allows to remove the unfavourable largest eigenvalues from the spectrum of the preconditioned coefficient matrix ${\mathbf{M}}^{ - 1} {\mathbf{C}}$ of ssSNPBLUP and of ssPCBLUP. However, the proposed definition of the deflation matrix did not affect, or very slightly, the rest of the spectrum of ssSNPBLUP, since the smallest eigenvalues remained similar among the different system matrices of ssSNPBLUP and of ssGBLUP. Similar results were obtained for ssPCBLUP. The deflation vectors spanned approximately the same space as the span of the eigenvectors corresponding to the largest eigenvalues of ${\mathbf{M}}^{ - 1} {\mathbf{C}}$ of ssSNPBLUP and of ssPCBLUP. The proposed definition of the deflation(-subspace) matrix allows smaller effective spectral condition numbers, and therefore better convergence, as expected from the theory. Decreasing the number of randomly chosen SNP effects per subdomain from 200 to 1 or 5 led to effective spectral condition numbers and convergence patterns for ssSNPBLUP similar to those for ssGBLUP. Based on our results and when fitting SNP effects, including 5 randomly chosen SNP effects per subdomain gave similar performance as including 1 SNP effect per subdomain. This similar performance depends probably on the properties of the genomic information, such as the amount of noise (or redundancy) in the genomic information, or the kind of effects fitted (e.g., SNP or PC effects). Indeed, when fitting PC effects, including 5 PC effects per subdomain gave worse performance than including 1 PC effect per subdomain, or even than including 5 SNP effects per subdomain (Table 4). It is worth noting that these results are interesting for ssSNPBLUP because they allow smaller Galerkin matrices, and therefore less memory use and lower computational costs. For ssPCBLUP, the size of the Galerkin matrices remained small thanks to the dimension reduction from SVD of ${\mathbf{Z}}$.

Regarding computational costs, the proposed definition of the deflation matrix was based on a subdomain deflation approach, allowing cheap and efficient computations [22]. Ideally, the deflation-subspace matrix ${\mathbf{Z}}_{d}$ should consist of eigenvectors associated with the unfavourable eigenvalues of ${\mathbf{M}}^{ - 1} {\mathbf{C}}$ [20–22]. However, the computation of these eigenvectors for large linear systems of equations can be very expensive, and these vectors might also be dense, leading to an increase of memory and expensive computations involving the deflation matrix ${\mathbf{P}}$. Therefore, defining sparse deflation vectors that approximate the same space as the span of the unfavourable eigenvalues of ${\mathbf{M}}^{ - 1} {\mathbf{C}}$ is desirable, and can be obtained with the subdomain deflation approach described by Frank and Vuik [22]. This approach gave good performance in several fields [22–24], and leads to interesting properties. The deflation-subspace matrix ${\mathbf{Z}}_{d}$ resulting from this approach is indeed sparse, is cheap to construct, involves a few additional and cheap computations, and has favourable properties for parallel computing [22]. For example, the deflation-subspace matrix in our implementation is stored as a vector of size of the number of equations of the system, and each entry of this vector contains the identification number of the subdomain associated with the corresponding equation. Moreover, the Galerkin matrix ${\mathbf{E}}$ was held in memory as a dense matrix in our implementation, which is possible on current shared-memory computers when the numbers of SNPs and traits are reasonable. Holding the Galerkin matrix in memory also allows efficient parallel computing using Intel MKL subroutines. Furthermore, while a suboptimal approach was used in this study to compute the Galerkin matrix ${\mathbf{E}}$, we expect its computation to be feasible within a limited amount of time and memory by taking further advantages of the symmetry of the coefficient matrix ${\mathbf{C}}$ and of the properties of the subdomain deflation approach.

Improvement of the current definition of the subdomains for ssSNPBLUP and ssPCBLUP could reduce further computational costs (i.e., time and memory requirements). The definition of subdomains used in this study was arbitrary, that is the number of SNP effects assigned to one subdomain was the same for each subdomain, and SNP effects assigned to one subdomain were randomly chosen. It would be worth investigating whether assignments of SNP effects to a subdomain based on properties of the SNP genotypes, such as linkage disequilibrium, could reduce the number of subdomains while maintaining, or decreasing, the obtained effective spectral condition numbers. Indeed, the current definition of subdomains could lead to too large Galerkin matrices for ssSNPBLUP with a large number of traits. Furthermore, for the field dataset, the current definition of subdomains did not allow to hold in memory the matrix ${\mathbf{CZ}}_{d}$ for computation efficiency [22]. Instead, we had to perform the multiplication of ${\mathbf{C}}$ by a vector twice each DPCG iteration, leading to double wall clock times per DPCG iteration in comparison to the PCG method.

For large datasets, a matrix-free approach (that is our second implementation) allows to solve ssSNPBLUP and ssPCBLUP with a (D)PCG method on current computers and with limited amounts of RAM and of wall clock time. Indeed, large and dense matrices of the linear system of Eq. (2), such as ${\mathbf{H}}^{13} = {\mathbf{G}}_{0}^{ - 1} \otimes {\mathbf{A}}^{ng} {\mathbf{Z}}$ for ssSNPBLUP, are never computed explicitly. Instead, the matrix-free approach takes advantage of the fact that the (D)PCG method requires the multiplication of ${\mathbf{C}}$ by a vector. For example, the multiplication of ${\mathbf{H}}^{13}$ by a vector ${\mathbf{d}}$ is performed in three parts, i.e. ${\mathbf{H}}^{13} {\mathbf{v}} = {\mathbf{G}}_{0}^{ - 1} \otimes \left[ {{\mathbf{A}}^{ng} \left[ {{\mathbf{Zd}}} \right]} \right]$ where the brackets $\left[ \cdot \right]$ indicate the order of the matrix–vector operations. Also, when using a matrix-free approach, one of the largest computational costs of ssSNPBLUP (ssPCBLUP) solved with a (D)PCG method is, most likely, the multiplication of ${\mathbf{Z}}$ (${\mathbf{T}}$) by a vector ${\mathbf{d}}$. Thus, it is expected that the main computational costs of ssSNPBLUP and of ssPCBLUP will increase linearly with increasing numbers of genotyped animals. Such a linear increase of the computational costs is also observed for ssGBLUP using APY [6] or ssGTBLUP based on a Woodbury decomposition of ${\mathbf{G}}$ [8].

While the solving process for ssPCBLUP seems to be more favourable than that for ssSNPBLUP in terms of memory and time requirements, the comparison between the two approaches should also consider additional computations, such as the SVD of the centered genotype matrix for ssPCBLUP. For the field dataset, the computational costs were quite substantial (i.e. > 15,000 s with 10 threads), and these will increase linearly with the number of genotyped animals and quadratically with the number of SNPs (assuming that the number of genotyped animals is larger than the number of SNPs). However, the time needed for SVD can be reduced by analysing different genome segments (e.g., chromosomes) in parallel [7]. For example, using an own Coarray Fortran program with 5 images (processes) using each 2 CPU, performing the SVD of 5 genome segments (of the same size) in parallel took 1276 s, and 16,662 PC were kept (instead of 13,803 PC kept with the SVD to the full genotype matrix). Performing the SVD on 5 genome segments instead of on the full genotype matrix, only marginally increased time and memory required for ssPCBLUP using the DPCG method with 1 PC per subdomain (results not shown). Therefore, further studies comparing computational costs for the whole process of ssPCBLUP, of ssSNPBLUP, but also of ssGBLUP and related methods (ssGBLUP using APY [6], and ssGTBLUP [8]) are needed. Such studies should consider costs of SVD, of computation of genomic relationship matrices, and of back-solving SNP effects from genomic estimated breeding values.

Because both PCG and DPCG methods are CG-based methods, the DPCG method can be easily implemented in current software based on the PCG method for other ssSNPBLUP (ssPCBLUP) models, or even for pedigree- and ssGBLUP models, for which convergence issues are observed. Modifications of existing PCG software would be mainly associated with the multiplication of ${\mathbf{P}} = {\mathbf{I}} - {\mathbf{CZ}}_{d} {\mathbf{E}}^{ - 1} {\mathbf{Z}}_{d}^{\varvec{'}}$ by a vector, which can rely on existing code for the multiplication of ${\mathbf{C}}$ by a vector. Using the DPCG method with pedigree-BLUP or ssGBLUP could also improve their convergence patterns. For example, the number of iterations to solve the pedigree-BLUP of the field dataset decreased by about 30% (in comparison to the PCG method) after associating one subdomain with each of the 100 sires that had the largest progeny. While this approach could not be generalised to other available field datasets (results not shown), it seems worthwhile to investigate the DPCG method in the pedigree- and ssGBLUP contexts for performing routine genetic evaluations with increasing datasets within a reasonable time.

## Conclusions

We showed that convergence issues observed with ssSNPBLUP and ssPCBLUP solved by the PCG method are related with larger eigenvalues and larger effective spectral condition numbers in comparison to ssGBLUP. These convergence issues of ssSNPBLUP and of ssPCBLUP were solved with a DPCG method, which is a two-level PCG method for ill-conditioned linear systems. As defined in this study, the DPCG method treats the largest unfavourable eigenvalues of the preconditioned coefficient matrix of ssSNPBLUP and of ssPCBLUP, and leads to a convergence pattern, which is at least similar to that of ssGBLUP.

## Additional files

**Additional file 1.** Derivation of a preconditioned deflated coefficient matrix. Description: Here we derive the preconditioned deflated coefficient matrix when the computational domain is divided such that some effects are included alone in a separate subdomain.

**Additional file 2:** **Figure S1.** Comparison of the estimates of ssGBLUP solved with the PCG method and of ssSNPBLUP solved with the DPCG method using five SNP effects per subdomain. Estimates are for all fixed effects and random additive genetic effects for the field dataset. **Figure S2.** Comparison of the estimates of ssGBLUP solved with the PCG method and of ssPCBLUP solved with the DPCG method using one PC effect per subdomain. Estimates are for all fixed effects and random additive genetic effects for the field dataset.
