# Supplementary material for: Deflated preconditioned conjugate gradient method for solving single-step BLUP models efficiently
Source: Genet Sel Evol. 2018 Nov 3;50:51. doi: 10.1186/s12711-018-0429-3 (PMC6215606; doi:10.1186/s12711-018-0429-3)
Supplement: Supplementary file 1 — Additional file 1. Derivation of a preconditioned deflated coefficient matrix. Description: Here we derive the preconditioned deflated coefficient matrix when the computational domain is divided such that some effects are included alone in a separate subdomain. [file 12711_2018_429_MOESM1_ESM.pdf]

Additional file 1: Derivation of a preconditioned deflated coefficient matrix

Here we derive the preconditioned deflated coefficient matrix when the computational domain is divided such that some effects are included alone in a separate subdomain.

Consider the system of equations $\mathbf{Cx} = \mathbf{b}$ with a symmetric positive semi-definite coefficient matrix $\mathbf{C} = \begin{bmatrix} \mathbf{C}_{11} & \mathbf{C}_{12} \\ \mathbf{C}_{21} & \mathbf{C}_{22} \end{bmatrix}$, a vector of solutions $\mathbf{x} = \begin{bmatrix} \mathbf{x}_1 \\ \mathbf{x}_2 \end{bmatrix}$, and a right-hand-side $\mathbf{b} = \begin{bmatrix} \mathbf{b}_1 \\ \mathbf{b}_2 \end{bmatrix}$. Consider also the deflation-subspace matrix $\mathbf{Z}_d = \begin{bmatrix} \mathbf{Z}_{d_{11}} & \mathbf{0} \\ \mathbf{0} & \mathbf{I}_z \end{bmatrix}$ where the submatrix $\mathbf{Z}_{d_{11}}$ contains only one non-zero element (which is equal to 1) per row, and the identity matrix $\mathbf{I}_z$ has the same size as $\mathbf{C}_{22}$.This deflation-subspace matrix $\mathbf{Z}_d$ corresponds to a division of a computational domain $\mathbb{R}^n$ (with $\mathbf{x} \in \mathbb{R}^n$) where each entry of $\mathbf{x}_2$ is included alone in a subdomain.

The deflation matrix $\mathbf{P}$ is defined as $\mathbf{I} - \mathbf{CZ}_d(\mathbf{Z}_d{}'\mathbf{CZ}_d)^{-1}\mathbf{Z}_d'$.

Using a block-wise inversion, it can be shown that:

$$(\mathbf{Z}_d{}'\mathbf{CZ}_d)^{-1} = \left(\begin{bmatrix} \mathbf{Z}_{d_{11}}{}' & \mathbf{0} \\ \mathbf{0} & \mathbf{I}_z \end{bmatrix}\begin{bmatrix} \mathbf{C}_{11} & \mathbf{C}_{12} \\ \mathbf{C}_{21} & \mathbf{C}_{22} \end{bmatrix}\begin{bmatrix} \mathbf{Z}_{d_{11}} & \mathbf{0} \\ \mathbf{0} & \mathbf{I}_z \end{bmatrix}\right)^{-1} =$$

$$\begin{bmatrix} \mathbf{Q} & -\mathbf{QZ}_{d_{11}}{}'\mathbf{C}_{12}\mathbf{C}_{22}^{-1} \\ -\mathbf{C}_{22}^{-1}\mathbf{C}_{21}\mathbf{Z}_{d_{11}}\mathbf{Q} & \mathbf{T} \end{bmatrix}$$

with $\mathbf{Q} = \left(\mathbf{Z}_{d_{11}}{}'\mathbf{C}^{11}\mathbf{Z}_{d_{11}} - \mathbf{Z}_{d_{11}}{}'\mathbf{C}_{12}\mathbf{C}_{22}^{-1}\mathbf{C}_{21}\mathbf{Z}_{d_{11}}\right)^{-1}$ and $\mathbf{T} = \mathbf{C}_{22}^{-1} + \mathbf{C}_{22}^{-1}\mathbf{C}_{21}\mathbf{Z}_{d_{11}}\mathbf{QZ}_{d_{11}}{}'\mathbf{C}_{12}\mathbf{C}_{22}^{-1}$

After some algebra, it follow that the preconditioned deflated coefficient matrix $\mathbf{M}^{-1}\mathbf{PC}$ is equal to:

$$\mathbf{M}^{-1}\mathbf{P}\mathbf{C} = \mathbf{M}^{-1}(\mathbf{I} - \mathbf{C}\mathbf{Z}_d(\mathbf{Z}_d{}'\mathbf{C}\mathbf{Z}_d)^{-1}\mathbf{Z}_d{}')\mathbf{C}$$

$$= \mathbf{M}^{-1}\begin{bmatrix}\mathbf{C}_{11} & \mathbf{C}_{12}\\ \mathbf{C}_{21} & \mathbf{C}_{22}\end{bmatrix}$$

$$- \mathbf{M}^{-1}\begin{bmatrix}\mathbf{C}_{11} & \mathbf{C}_{12}\\ \mathbf{C}_{21} & \mathbf{C}_{22}\end{bmatrix}\begin{bmatrix}\mathbf{Z}_{d_{11}} & \mathbf{0}\\ \mathbf{0} & \mathbf{I}\end{bmatrix}\begin{bmatrix}\mathbf{Q} & -\mathbf{Q}\mathbf{Z}_{d_{11}}{}'\mathbf{C}_{12}\mathbf{C}_{22}^{-1}\\ -\mathbf{C}_{22}^{-1}\mathbf{C}_{21}\mathbf{Z}_{d_{11}}\mathbf{Q} & \mathbf{T}\end{bmatrix}\begin{bmatrix}\mathbf{Z}_{d_{11}}{}' & \mathbf{0}\\ \mathbf{0} & \mathbf{I}\end{bmatrix}\begin{bmatrix}\mathbf{C}_{11} & \mathbf{C}_{12}\\ \mathbf{C}_{21} & \mathbf{C}_{22}\end{bmatrix}$$

$$= \mathbf{M}^{-1}\begin{bmatrix}\mathbf{C}_{11} & \mathbf{C}_{12}\\ \mathbf{C}_{21} & \mathbf{C}_{22}\end{bmatrix}$$

$$- \mathbf{M}^{-1}\begin{bmatrix}\left(\mathbf{C}_{11}\mathbf{Z}_{d_{11}}\mathbf{Q} - \mathbf{C}_{12}\mathbf{C}_{22}^{-1}\mathbf{C}_{21}\mathbf{Z}_{d_{11}}\mathbf{Q}\right)\mathbf{Z}_{d_{11}}{}'\mathbf{C}_{11} + \left(\mathbf{C}_{12}\mathbf{T} - \mathbf{C}_{11}\mathbf{Z}_{d_{11}}\mathbf{Q}\mathbf{Z}_{d_{11}}{}'\mathbf{C}_{12}\mathbf{C}_{22}^{-1}\right)\mathbf{C}_{21} & \mathbf{C}_{12}\\ \mathbf{C}_{21} & \mathbf{C}_{22}\end{bmatrix}$$

$$= \mathbf{M}^{-1}\begin{bmatrix}\mathbf{C}_{11} - \left(\mathbf{C}_{11}\mathbf{Z}_{d_{11}}\mathbf{Q} - \mathbf{C}_{12}\mathbf{C}_{22}^{-1}\mathbf{C}_{21}\mathbf{Z}_{d_{11}}\mathbf{Q}\right)\mathbf{Z}_{d_{11}}{}'\mathbf{C}_{11} + \left(\mathbf{C}_{12}\mathbf{T} - \mathbf{C}_{11}\mathbf{Z}_{d_{11}}\mathbf{Q}\mathbf{Z}_{d_{11}}{}'\mathbf{C}_{12}\mathbf{C}_{22}^{-1}\right)\mathbf{C}_{21} & \mathbf{0}\\ \mathbf{0} & \mathbf{0}\end{bmatrix}$$

$$= \mathbf{M}^{-1}\begin{bmatrix}\mathbf{S} - \mathbf{S}\mathbf{Z}_{d_{11}}\left(\mathbf{Z}_{d_{11}}{}'\mathbf{S}\mathbf{Z}_{d_{11}}\right)^{-1}\mathbf{Z}_{d_{11}}{}'\mathbf{S} & \mathbf{0}\\ \mathbf{0} & \mathbf{0}\end{bmatrix}$$

with $\mathbf{S} = \mathbf{C}_{11} - \mathbf{C}_{12}\mathbf{C}_{22}^{-1}\mathbf{C}_{21}$.

This development proofs that the division of the ssSNPBLUP domain with 1 SNP effect per subdomain leads to a system matrix $\mathbf{M}^{-1}\mathbf{P}\mathbf{C}$ with zero entries for all equations associated with the SNP effects.
