# Supplementary material for: Deflated preconditioned conjugate gradient method for solving single-step BLUP models efficiently
Source: Genet Sel Evol. 2018 Nov 3;50:51. doi: 10.1186/s12711-018-0429-3 (PMC6215606; doi:10.1186/s12711-018-0429-3)
Supplement: Supplementary file 2 — Additional file 2: Figure S1. Comparison of the estimates of ssGBLUP solved with the PCG method and of ssSNPBLUP solved with the DPCG method using five SNP effects per subdomain. Estimates are for all fixed effects and random additive genetic effects for the field dataset. Figure S2. Comparison of the estimates of ssGBLUP solved with the PCG method and of ssPCBLUP solved with the DPCG method using one PC effect per subdomain. Estimates are for all fixed effects and random additive genetic effects for the field dataset. [file 12711_2018_429_MOESM2_ESM.pdf]

Additional file 2

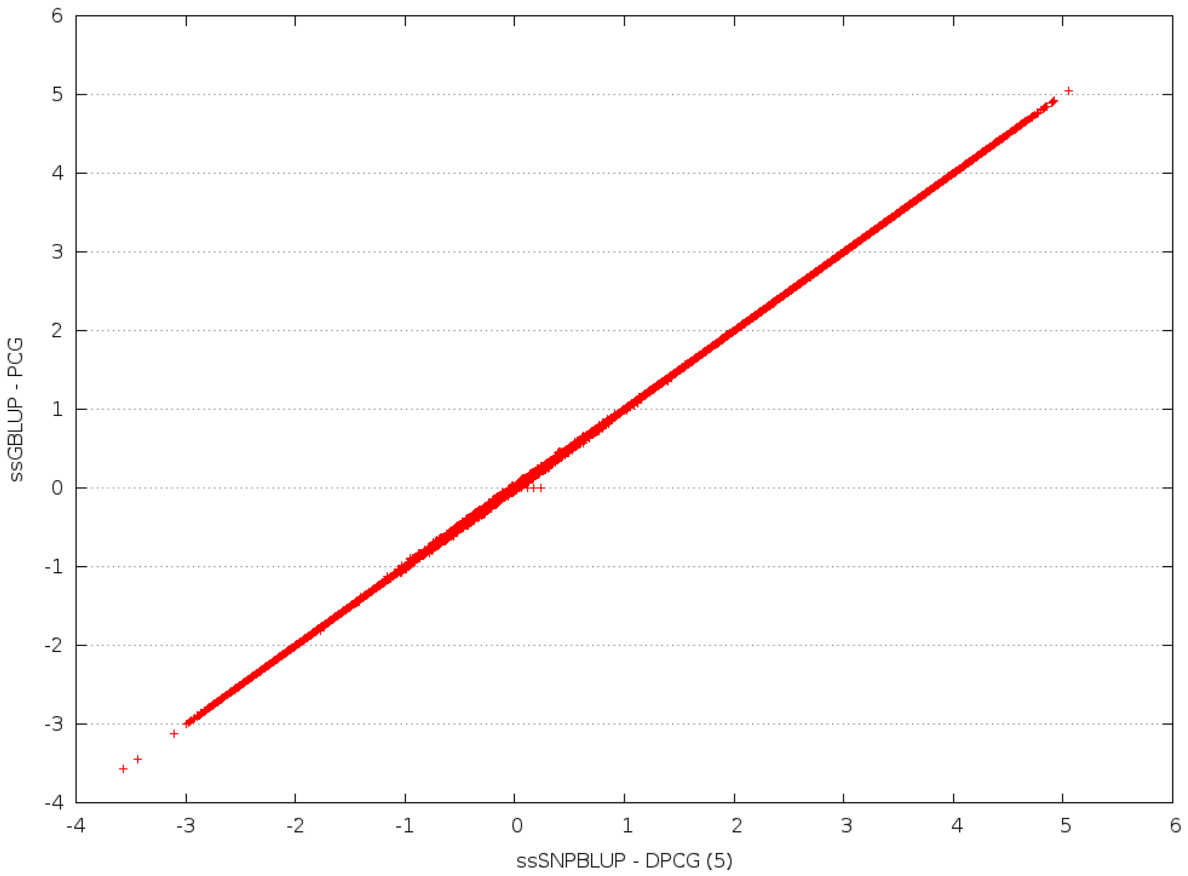

Figure S1. Comparison of the estimates of ssGBLUP solved with the PCG method and of ssSNPBLUP solved with the DPCG method using 5 SNP effects per subdomain. Estimates are for all fixed effects and random additive genetic effects for the field dataset.

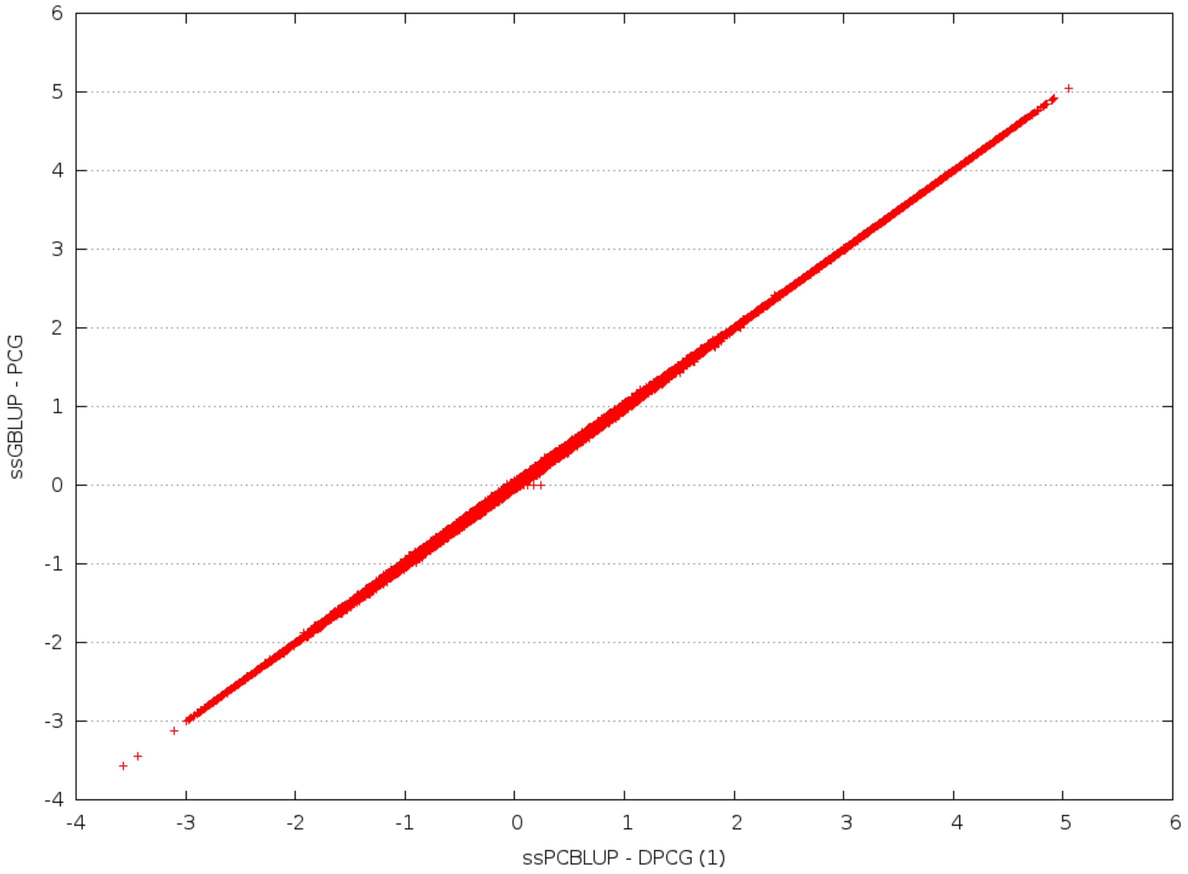

Figure S2. Comparison of the estimates of ssGBLUP solved with the PCG method and of ssPCBLUP solved with the DPCG method using 1 PC effect per subdomain. Estimates are for all fixed effects and random additive genetic effects for the field dataset.
